# A unified model of transient poration induced by antimicrobial peptides

**DOI:** 10.1073/pnas.2510294122

**Published:** 2025-08-29

**Authors:** Amy Rice, Andriana C. Zourou, Myriam L. Cotten, Richard W. Pastor

**Affiliations:** ^a^Laboratory of Computational Biology, National Heart, Lung, Blood Institute, National Institutes of Health, Bethesda, MD 20892; ^b^Department of Applied Science, William & Mary, Williamsburg, VA 23185; ^c^Department of Biochemistry and Biophysics, Oregon State University, Corvallis, OR 97331

**Keywords:** antimicrobial peptides, cell-penetrating peptides, asymmetric lipid bilayers, membrane active peptides

## Abstract

Membrane active peptides (MAPs) represent a diverse group of agents that disrupt the integrity of lipid membranes. One class of MAPs, antimicrobial peptides (AMPs), destroy bacteria by transiently porating the bacterial membrane causing leakage of cellular contents. Transient leakage is classified as “graded,” where all vesicles in a population leak partially, or “all-or-none,” where some vesicles leak completely. However, the molecular interactions underlying transient leakage have eluded experimental determination. Here, dye leakage experiments with the AMP piscidin 1 (P1) show that graded leakage can be converted to all-or-none by simply adding a defect-promoting lysophospholipid. Molecular dynamics simulations demonstrate that area stress arising from membrane asymmetry decreases the energy of pore formation and is highly lipid dependent. Furthermore, lipids and peptides translocate the bilayer through these pores, leading to area-relaxed states where poration is highly unfavorable. Even pores too small to leak dye relieve area stress; they are the “none” component of all-or-none release. These observations lead to development of a quantitative model where graded and all-or-none leakage are treated as a continuum explained by a single mechanism that accounts for the local peptide concentration and probabilities of different pore sizes. This unified model accurately reproduces the P1 dye leakage data and provides an explanation for varying pore energy, size, and probability within the framework of asymmetry-driven poration. This model is expected to be applicable to other MAPs, including cell-penetrating peptides, and could provide a framework for designing peptides with greater cellular specificity, a long-sought outcome.

## Significance

Membrane active peptides are cationic amphipathic molecules that disrupt biological membranes to perform their function. Antimicrobial peptides, which are permeabilizing, kill bacteria and cancer cells, making them promising candidates to fight drug-resistance; cell-penetrating peptides cross membranes without permeabilization, and thus can deliver drugs intracellularly. When added to solute-containing vesicles that mimic cells, membrane active peptides cross membranes until area-imbalance is dissipated. During this transient process, leakage can occur, differing in duration and extent of solute loss, and modulated by membrane composition and peptide sequence. To explain these variations, we present a mechanistic framework based on the structural states adopted by antimicrobial peptides during membrane crossing. With this insight, membrane active peptides could be tailored to target cells with enhanced specificity.

Membrane active peptides (MAPs) are a broad class of biological peptides that interact with and alter lipid membranes. These interactions include disruption of bacterial cell membranes leading to cell death by antimicrobial peptides (AMPs) as well as nondestructive membrane translocation by cell-penetrating peptides (CPPs) to deliver cargo. Due to these myriad effects, MAPs are of broad therapeutic interest for development of novel antibacterials (AMPs) and targeted drug or gene delivery (CPPs) (1–8). However, while AMPs and CPPs have been studied for several decades, the molecular structures and states they adopt to permeabilize lipid membranes remain debated (4, 9–13), hampering efforts to rationally design enhanced analogs.

A hallmark of AMPs is their transient and incomplete membrane poration behavior, explaining the challenges associated with mechanistic studies (4, 14–18). In contrast to water-filled channels that form stable transmembrane pores and induce vesicular leakage at lipid-to-peptide ratios (L/P) above 1,000:1, AMPs typically require higher peptide concentrations (L/P < 500:1) to be active (16). Furthermore, their leakage qualifies as incomplete when compared to that of stable channels, which can release the entire content (e.g., hundreds of dye molecules) of large unilamellar vesicles (LUVs) in a few tenths of a second (19, 20). The time course of transient dye release by AMPs is characterized by an initial rapid burst of dye release in the first few minutes followed by a decline or stop, often before all of the vesicle contents have been released; adding additional peptide causes a new burst of dye release (16, 21). Because of its nonequilibrium nature, this type of transient leakage cannot be adequately modeled or understood through an equilibrium description. Considering only the peptide concentration or L/P ratio, for example, cannot explain why permeabilization stops after some amount of time, or how it is able to be restarted.

The transience of poration is believed to be caused by an initial imbalance or asymmetry (in charge, mass, surface tension, or some other property) across the membrane created by the peptide, with poration or loss of membrane integrity occurring as the peptides equilibrate across the membrane. This asymmetry-driven mechanism for transient leakage was first hypothesized in 1995, when Matsuzaki et al. (22) ascribed the dissipation of magainin 2 induced leakage to peptide translocation across the membrane; once the asymmetry is relaxed, the leakage stops. Since then, asymmetry stress has been widely accepted to be the driving force of poration through a self-dissipating process by which poration relaxes the initial asymmetry and favors pore closure (4, 18, 23, 24). This mechanism has been further bolstered by observations of coupled vesicle permeabilization and peptide translocation for a number of different transient permeabilizers (22, 25, 26).

**Fig. 1.**
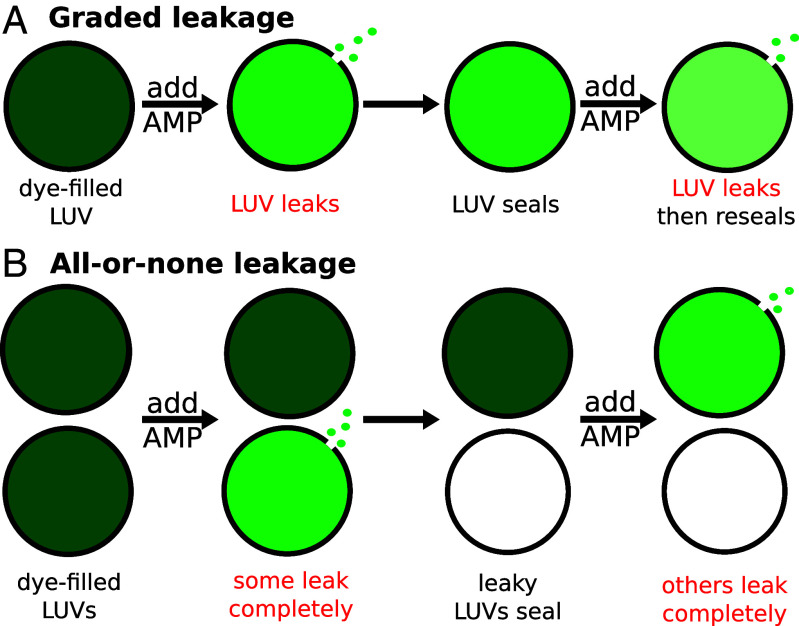
Idealized schematics of (*A*) graded leakage and (*B*) all-or-none leakage. The response of vesicles (circles) containing trapped dye (green shading) is depicted.

Based on experimental observations, AMPs that induce transient leakage appear to operate through one of two idealized vesicular outcomes: “graded,” where all of the vesicles release some of their contents (Fig. 1*A*), or “all-or-none,” where some of the vesicles release all their contents and the others have no leakage (Fig. 1*B*) (4, 18). One of the main assays used to distinguish between all-or-none and graded leakage is the requenching experiment designed by Wimley et al. (27–29). A fluorescent dye is entrapped in vesicles together with a quencher, resulting in an initial internal quenching value, $Q_{\mathrm{in}}$. Following the addition of an AMP at a chosen L/P ratio, the fraction of dye released, $f_{\mathrm{out}}$, is titrated with the quencher, so that the amount of quenching arising from dye remaining within the LUVs can be assessed. This process is repeated using a broad range of L/P values, as needed to generate a plot of $Q_{\mathrm{in}}$ as a function of $f_{\mathrm{out}}$. A constant value of $Q_{\mathrm{in}}$ vs. $f_{\mathrm{out}}$ indicates that the leakage is all-or-none while a monotonically increasing value corresponds to graded leakage. However, the binary of graded or all-or-none oversimplifies the diverse range of experimental leakage behaviors observed between these two extremes, which are more accurately explained as a continuum of leakage behaviors (23, 30, 31). In this combined simulation and experimental study, we confirm that graded and all-or-none leakage are a spectrum, and that both extremes can be explained by a single mechanism that accounts for the molecular interactions underlying transient leakage.

There are several mathematical models to describe graded and all-or-none leakage (28, 29, 32), as well as qualitative models. In particular, Rathinakumar and Wimley (20) proposed that an alternate pathway for membrane stress equilibration could account for the nonleaky vesicles in all-or-none leakage; this alternate pathway was suggested to occur via a mechanism similar to translocation by CPPs, which can translocate membranes without releasing material from cells. Wheaten et al. (26) later directly observed such a leakage-free translocation pathway in fluorescence microscopy experiments, noting that peptide translocation sometimes precedes dye leakage rather than following it, a phenomenon they referred to as “silent” leakage. Additionally, high temperature simulations by Ulmschneider (13) of the AMP PGLa demonstrated its ability to translocate through a bilayer one peptide at a time on the µs timescale. The present work adopts the designation of Rathinakumar and Wimley (20) of “CPP-like” for small pores that translocate lipids and peptides from one leaflet to the other, relieving asymmetry stress without measurable dye release.

Regardless of the leakage mechanism, elucidating transient leakage requires understanding and modeling membrane asymmetry since under experimental conditions peptides are initially only on the outside of vesicles in an asymmetric distribution, and this asymmetry is believed to drive transient poration. Asymmetry can take two forms, which are neither mutually exclusive nor entirely independent: compositional asymmetry (different lipid or protein species in each leaflet) and area asymmetry (different numbers of proteins or lipids in each leaflet). Both types of asymmetry affect bulk bilayer properties (33–36). Molecular dynamics (MD) simulations of asymmetric bilayers have typically sought to impose compositional asymmetry while minimizing the effects of area asymmetry (36–38). However, lipid flip-flop is very slow when compared to simulation and experimental timescales, thus relieving leaflet area imbalances may not always be achievable or desirable when modeling asymmetric membranes.

The effects of area stress on membrane structure were demonstrated in our recent work (39) (hereafter referred to as Paper 1), which featured piscidin 1 (P1), an archetypal cationic, amphipathic AMP derived from fish. P1 (sequence: FFHHIFRGIVHVGKTIHRLVTG-NH_2_), which has a net charge of +4 at neutral pH, is active on drug-resistant bacteria and has been thoroughly characterized on a biophysical level for its structural features and membrane active properties (40–47). In that earlier work, MD simulations examined asymmetrically distributed P1 both with and without first relaxing the membrane area stress, and showed that membrane defects were significantly more frequent in the systems with area stress. The present study builds directly on Paper 1, combining fluorescent dye leakage experiments, MD simulations, and mathematical modeling to probe in more detail the pores induced by P1 in terms of their structural characteristics and leakage mechanism, and to explore the role that asymmetry stress plays. Importantly, the effects of two different lipid compositions are compared, 3:1 POPC:POPG; and 2:1:1 POPC:POPG:(16:0 lysoPC).

Two types of experiments were performed. 1) “Start-stop” dye leakage assays, where a time course of fractional leakage is assessed over a population of LUVs to confirm that P1-induced leakage is transient and can be restarted by adding more P1, as well as to determine leakage rates over a wide range of L/P ratios. 2) Fluorescence requenching assays, which measure the degree of quenching inside a population of vesicles that have partially released their contents, to resolve graded vs. all-or-none apparent leakage behaviors in two different lipid compositions.

Additionally, three distinct sets of MD simulations were carried out. 1) Conventional MD simulations of membrane-bound P1 to identify P1-induced defects, visualize pore structure as a function of leakage mechanism, and quantify defect rates under conditions of either symmetry (area-relaxed, AR) or high area stress (area-stressed, AS). 2) Biased free energy simulations to rigorously calculate potentials of mean force (PMFs), or energy landscapes, of pore formation with and without area stress, thereby enabling examination of how asymmetry stress alters the probability of pore formation and closure. 3) Conventional MD simulations of both CPP-like and fully hydrated pores generated by the preceding PMFs, with or without area stress, to explore the time evolution of pores as lipids and peptides translocate and pores close. Effects of asymmetry loss, lipid composition, and peptide inhomogeneity were also considered.

While fluorescence requenching experiments demonstrated a change in P1 leakage mode from graded to all-or-none when lysoPC was added, MD simulations showed no change in P1 poration mechanism in the presence of lysoPC. Taken together, these results motivated development of a unified model for transient leakage that describes graded and all-or-none leakage as a single mechanism rather than two distinct processes, and that can be used to fit the experimental requenching data while recapitulating the molecular interactions underlying transient leakage. In this unified view of leakage, asymmetry stress can be relieved through either 1) CPP-like intermediates that do not release dye or 2) fully hydrated metastable pores that enable dye leakage, with the competition between the two pathways determining whether vesicles appear to leak or not. The leakage behavior appears graded or all-or-none, depending on the kinetics of pore formation, pore closure, and dye leakage; these quantities are modulated by the lipid composition, the extent of area stress, as well as the peptide concentration and inhomogeneity of its distribution on the membrane surface. Since AMPs are not the only peptides that induce transient leakage, this model is broadly applicable to many classes of MAPs. It also provides principles that could be used to design peptides that have enhanced specificity to selected cells based on their membrane compositions.

## Results

### Leakage Induced by Piscidin 1 Is Transient.

**Fig. 2.**
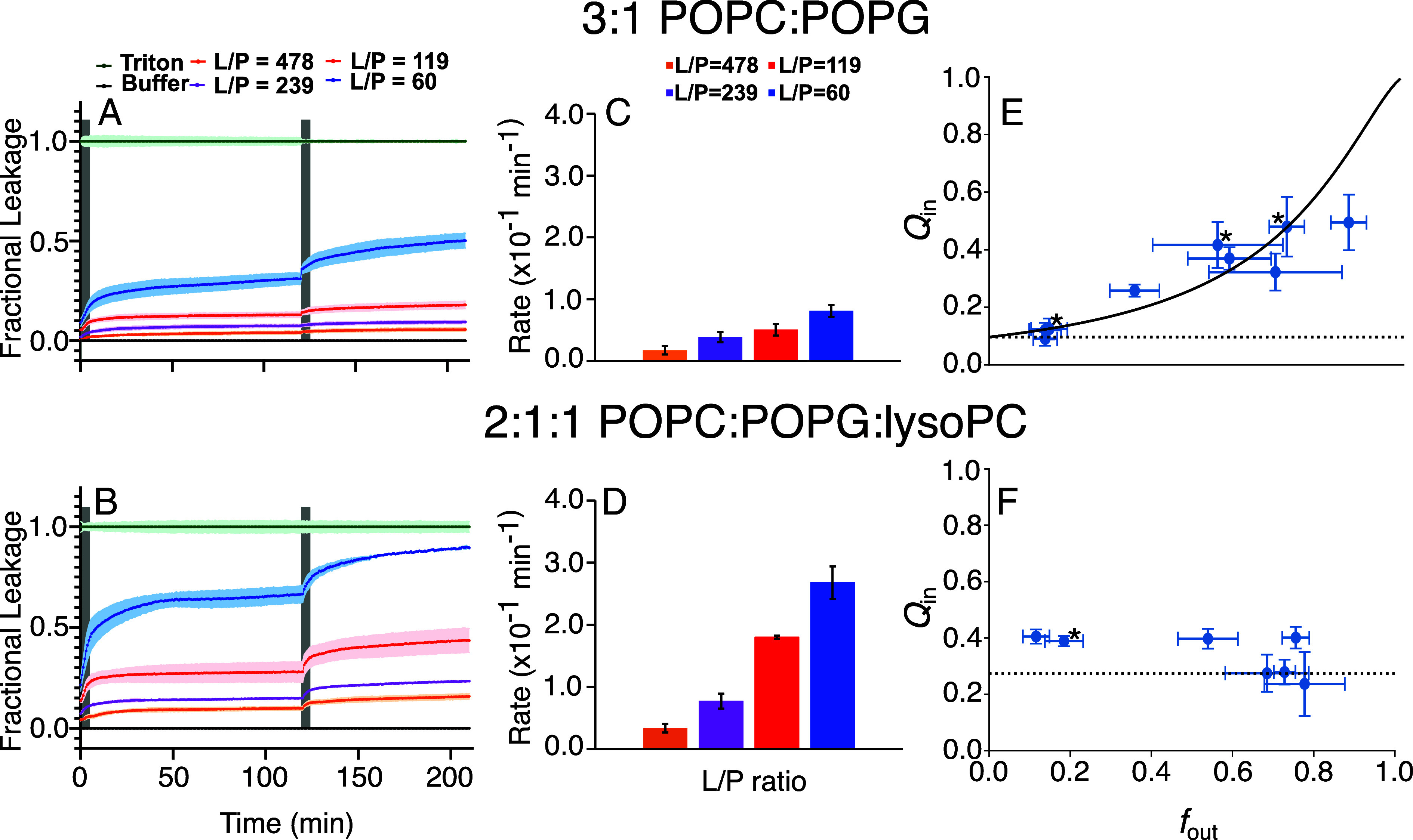
Fluorescence assays in 3:1 POPC:POPG and 2:1:1 POPC:POPG:lysoPC. (*A* and *B*) Fractional leakage observed over time after adding P1 to calcein-loaded LUVs in two additions, at lipid-to-peptide ratios (L/P) = 478, 239, 119, and 60. The gray-shaded regions crossing the curves indicate when the P1 additions were made. Vesicles treated with Triton-X and buffer were used as positive and negative controls, respectively. Shaded colored regions around the plots correspond to the SD. (*C* and *D*) Rate of fractional leakage change after each P1 addition, estimated from the slopes of curves collected individually for each L/P. Error shown is the SEM. (*E* and *F*) Change in internal quenching $Q_{\mathrm{in}}$ as a function of fractional leakage $f_{\mathrm{out}}$. All data points were collected in triplicate, except those denoted by an asterisk which were collected in duplicate. The error shown corresponds to the SD of the multiple measurements. The horizontal dashed lines demonstrate ideal all-or-none behavior, while the solid curve for POPC:POPG shows ideal graded behavior with a slight preference for release of DPX over ANTS (α=1.43, where α is the ratio of DPX and ANTS release rates).

Start-stop dye leakage assays were performed to gain insights into the kinetics of the leakage induced by P1 acting on 3:1 POPC:POPG and 2:1:1 POPC:POPG:lysoPC LUVs. The peptide was tested at four different L/Ps bracketing its EC_50_ values, which were previously determined in Paper 1 and confirmed here (*SI Appendix*, Table S1). As displayed in Fig. 2 *A* and *B*, after adding the peptide to the LUVs, the fluorescence was monitored continuously, revealing a burst phase followed by the leveling of the fluorescence within an hour. When P1 was reapplied at the same L/P an hour later, a second burst was detected, albeit at a lower amplitude than the first one.

For a deeper analysis, the rate of each burst phase was extracted by quantifying its slope, shown in Fig. 2 *C* and *D*. In both lipid compositions, the first-burst data demonstrate that increasing the peptide concentration (i.e., decreasing L/P) correlates with a faster burst rate and time to reach plateau. For the second burst, the transmembrane asymmetry is smaller, producing a much weaker effect to the point that the different L/P values become statistically indistinguishable. Strikingly, the rates are much slower in POPC:POPG than in POPC:POPG:lysoPC. For instance, at L/P = 60, the rate of the first burst was 3.3-fold higher in POPC:POPC:lysoPC (2.7 × 10^−1^ min^−1^) than in POPC:POPG (8.1 × 10^−2^ min^−1^). The data shown in *SI Appendix*, Fig. S1 support the result that the rate constants as a function of concentration are correspondingly higher for the lysoPC-containing bilayers. POPC:POPG lacks the lysolipid that increases the number of defects in the membrane, as shown in Paper 1. Hence, these results clearly highlight a correlation between the rate of leakage (and therefore the relative rates of pore opening and closing) and the number of defects in the membrane (*SI Appendix*, section S1.1 and Fig. S2).

### Piscidin 1 Can Induce Graded or Predominantly All-or-None Leakage.

The fluorescence requenching assay was performed to determine the mode of leakage when P1 is added 3:1 POPC:POPG and 2:1:1 POPC:POPG:lysoPC LUVs. Fig. 2 *E* and *F* displays the plots of $Q_{\mathrm{in}}$ (the normalized fluorescence of ANTS inside the vesicles) vs. $f_{\mathrm{out}}$ (fraction of released ANTS) that were obtained for the two lipid systems. As described in the Introduction, when $f_{\mathrm{out}}$ increases, $Q_{\mathrm{in}}$ remains constant in the all-or-none mode of leakage since the vesicles are either intact or completely empty. In contrast, the graded mode of leakage gives rise to values of $Q_{\mathrm{in}}$ that increase since all vesicles partially release not only the dye but also the quencher.

For 3:1 POPC:POPG LUVs, the data show a straightforward behavior. Within error, $Q_{\mathrm{in}}$ increases across the range of $f_{\mathrm{out}}$ values, consistent with graded leakage. As expected, the $Q_{\mathrm{in}}$ error bars tend to increase at higher peptide concentrations since fewer vesicles contain dye, and thus their fluorescence contribution to the $Q_{\mathrm{total}}$ used to calculate $Q_{\mathrm{in}}$ decreases (28). Despite the error bars, it is clear that the leakage is graded. The best fit of the data to the equation for ideal graded leakage (*SI Appendix*, Eq. **S8**) yields [DPX]_0_ = 0.011 ± 0.002 mM and α=1.43±0.27, indicating a slight preference for release of DPX over ANTS (28, 29). A completely different behavior is observed in 2:1:1 POPC:POPG:lysoPC LUVs (Fig. 2*F*). While $Q_{\mathrm{in}}$ is roughly constant for 0.15 $<f_{\mathrm{out}}<$ 0.4, the values then drop at larger $f_{\mathrm{out}}$. Overall, P1 acts in a predominantly all-or-none fashion, displaying a tendency to rise at lower concentrations but having an all-or-none behavior at higher concentrations.

### Pores Form More Easily and Are More Stable in AS Systems.

**Fig. 3.**
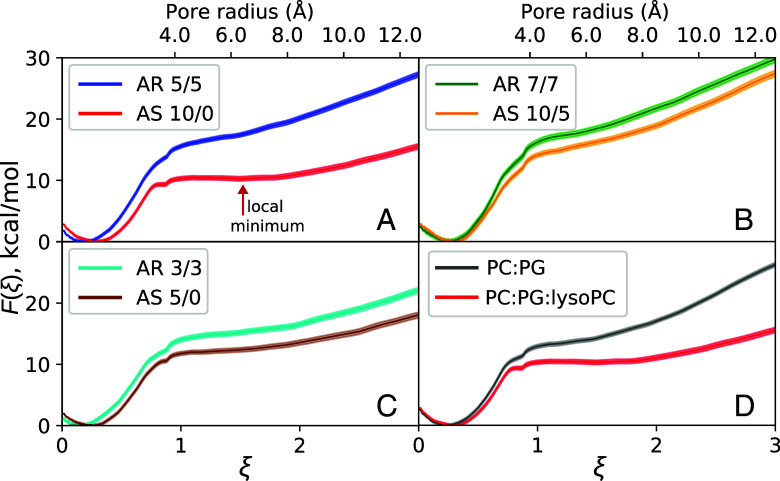
Free energy F(ξ) of pore formation and expansion in AS and AR systems. Values of the pore reaction coordinate ξ < 1 correspond to pore initiation, while values ≥ 1 correspond to pore expansion and can be converted to an approximate pore radius by multiplying ξ by 4.22 Å. Error in each free energy profile is represented by the shaded region. Comparison between (*A*) AR 5/5 and AS 10/0 systems, (*B*) AR 7/7 and AS 10/5 systems, (*C*) AR 3/3 and AS 5/0 systems, and (*D*) AS 10/0 in 3:1 POPC:POPG (gray data) and 2:1:1 POPC:POPG:lysoPC (red data). The arrow in panel *A* indicates the local minimum of the AS 10/0 energy landscape.

Paper 1 proposed defects as progenitors of transient pores but lacked data definitively linking defects to poration. PMF, or free energies, of pore formation were calculated here to directly determine the effects of area stress and peptide asymmetry on poration. The energy of pore formation was calculated as a function of the extended pore reaction coordinate ξ. As hypothesized, the energy required both to initiate a pore and to expand the pore radius is larger in the AR symmetric systems than in the paired AS systems (Fig. 3). Additional PMF calculations performed on an area relaxed system with a 10/0 peptide distribution demonstrate that area stress, not peptide asymmetry, accounts for most of the poration energy differences observed between AS and symmetric systems (*SI Appendix*, Fig. S3 and section S1.2).

Poration free energies from the five independent runs of each 2:1:1 POPC:POPG:lysoPC system are shown in *SI Appendix*, Fig. S4, while the average poration free energy profile for each system are presented in Fig. 3. All of the free energy profiles have a global minimum around ξ∼0.25, corresponding to a flat, unperturbed bilayer. Values less than this represent conformations where water is excluded from the bilayer headgroup region and are energetically unfavorable. The location of this minimum is shifted slightly lower in the AR systems than in the AS ones because thickness fluctuations are more pronounced under conditions of area stress (*SI Appendix*, section S1.1 and Table S2). As a check, the energy of defect formation can be estimated both from the free energy profiles as well as from unbiased simulations, with the two independent methods showing excellent agreement (*SI Appendix*, section S1.3 and Fig. S5).

All six free energy profiles display a small shoulder at ξ∼0.87, which corresponds to formation of a membrane-spanning water wire. Formation of this water wire is 4.5 kcal/mol more favorable in AS 10/0 than in AR 5/5 (*SI Appendix*, Table S3); the same trend holds for the other two L/P ratios simulated. CPP-like pores (ξ∼1.18) similarly have lower free energy barriers in AS than AR systems (*SI Appendix*, Table S3), and the energy of pore expansion is also significantly larger in AS systems. For example, the energy needed to generate a 10 Å radius pore is 10.3kcal/mol lower in the AS 10/0 peptide distribution (12.4 kcal/mol) than in the symmetric AR 5/5 system (22.7 kcal/mol). Smaller energy differences of 2.8 and 3.5 kcal/mol are observed for the other two L/P ratios, which both have a peptide number asymmetry δ=5 and therefore a lower extent of area mismatch between the AS and AR configurations.

There are important differences in the shapes of the energy landscapes between AS and AR systems. The AS 10/0 free energy profile displays a shallow local minimum at ξ=1.51 and a plateau in the range of ξ=1.11 to 1.83 corresponding to a metastable pore with a radius of 4.7 to 7.7 Å and energy of ∼10.3 kcal/mol. Similarly, the AS 5/0 system has a plateau at ξ=1.11 to 1.52 corresponding to a pore radius of 4.7 to 6.5 Å. None of the free energy profiles calculated for AR systems show local minima or plateaus, indicating no metastable pore formation.

### Lipid Composition Influences AS 10/0 Poration Free Energy Without Altering the Poration Pathway.

To examine the differences in poration free energy between 2:1:1 POPC:POPG:lysoPC and 3:1 POPC:POPG, free energy profiles for pore formation in 3:1 POPC:POPG with an AS 10/0 peptide distribution were calculated. Fig. 3*D* compares the free energy of pore formation and expansion for an AS 10/0 distribution in both lipid compositions. Compared to 2:1:1 POPC:POPG:lysoPC, the energy needed to form a water wire, a CPP-like pore, or a fully hydrated 10 Å radius pore are higher in 3:1 POPC:POPG: 11.3 ± 0.3 vs. 9.3 ± 0.3 kcal/mol for water wire formation, 13.3 ± 0.3 vs. 10.4 ± 0.3 kcal/mol for CPP-like pore formation, and 20.2 ± 0.3 vs. 12.4 ± 0.4 kcal/mol for formation of a 10 Å radius pore. The free energy landscape in 3:1 POPC:POPG has a much smaller plateau region, extending from ξ=1.1 to 1.3, indicating a smaller radius metastable state. Additionally, for large ξ the free energy increases more steeply in 3:1 POPC:POPG than in any of the 2:1:1 POPC:POPG:lysoPC landscapes. This is because, for large pore radii, the energy is dominated by the line tension of the pore rim; line tension is related to the spontaneous curvature of the membrane, with line tension increasing as the spontaneous curvature becomes more negative (48). Lysolipids induce positive spontaneous curvature, so the line tension of the pore is smaller when lysoPC is present in the bilayer and the poration free energy rises less steeply as a result. However, despite the experimental differences in leakage behavior (graded vs. all-or-none), both the free energy landscapes and poration pathway are qualitatively similar to one another.

### Differences in Peptide Insertion Explain Variability in Pore Stability.

**Fig. 4.**
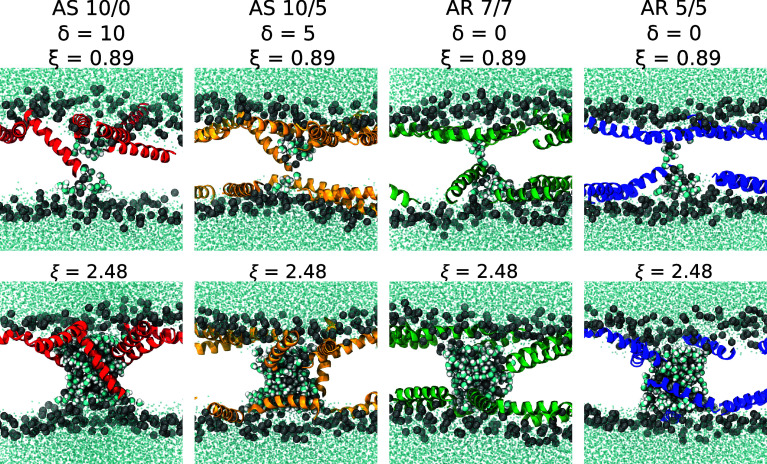
Representative snapshots with pore reaction coordinate ξ=0.89 and ξ=2.48 for four AS or AR systems, with the peptide number asymmetry δ indicated. Bulk water depicted as cyan dots, pore water molecules are cyan/white spheres, lipid phosphorus atoms are gray spheres, and peptides are colored helical ribbons.

The differences in pore energy and stability between AS and AR systems can be explained in part by analyzing the behavior of peptides within the pore. As evident in Fig. 4, peptides in AS systems are more likely to tilt deeply into the membrane, past the bilayer midplane as the pores initiate (top row), and become transmembrane as the pore grows (bottom row). In contrast, peptide termini in the AR systems rarely insert past the bilayer midplane and have fewer transmembrane states.

This difference in peptide behavior can be seen in two ways. First, for four of the systems, the maximum insertion depth of the peptide termini was calculated over all umbrella sampling (US) windows and plotted as a function of ξ (*SI Appendix*, Fig. S6*A*). Additionally, the average number of inserted peptides was calculated as a function of ξ (*SI Appendix*, Fig. S6*B*), where a peptide was considered “inserted” if one terminus extended past the bilayer midplane position. For small values of ξ (< 0.5) there is little difference between systems. Water wire formation and initial pore nucleation, however, is accompanied by much deeper peptide insertion in AS 10/0 (red data) and AS 10/5 (yellow) than in the two AR systems (green and blue). This trend continues and becomes more pronounced for larger ξ as the pore radius grows.

This contrasting peptide behavior can be rationalized as arising from the area stress within the bilayer. In particular, tilting or insertion of a peptide from surface-bound to a transmembrane state greatly reduces the cross-sectional area of the peptide within the overcrowded leaflet, and represents one way for area stress to be partially relaxed. A similar phenomenon was observed in a study by Park et al. (33) where the tilt angle of WALP23 changed with increasing bilayer area mismatch and the center of mass of the transmembrane peptide shifted 2 to 4 Å past the bilayer midplane in systems with large area mismatch.

### Lipids Translocate Through Metastable AS Pores While AR Pores Quickly Close.

**Fig. 5.**
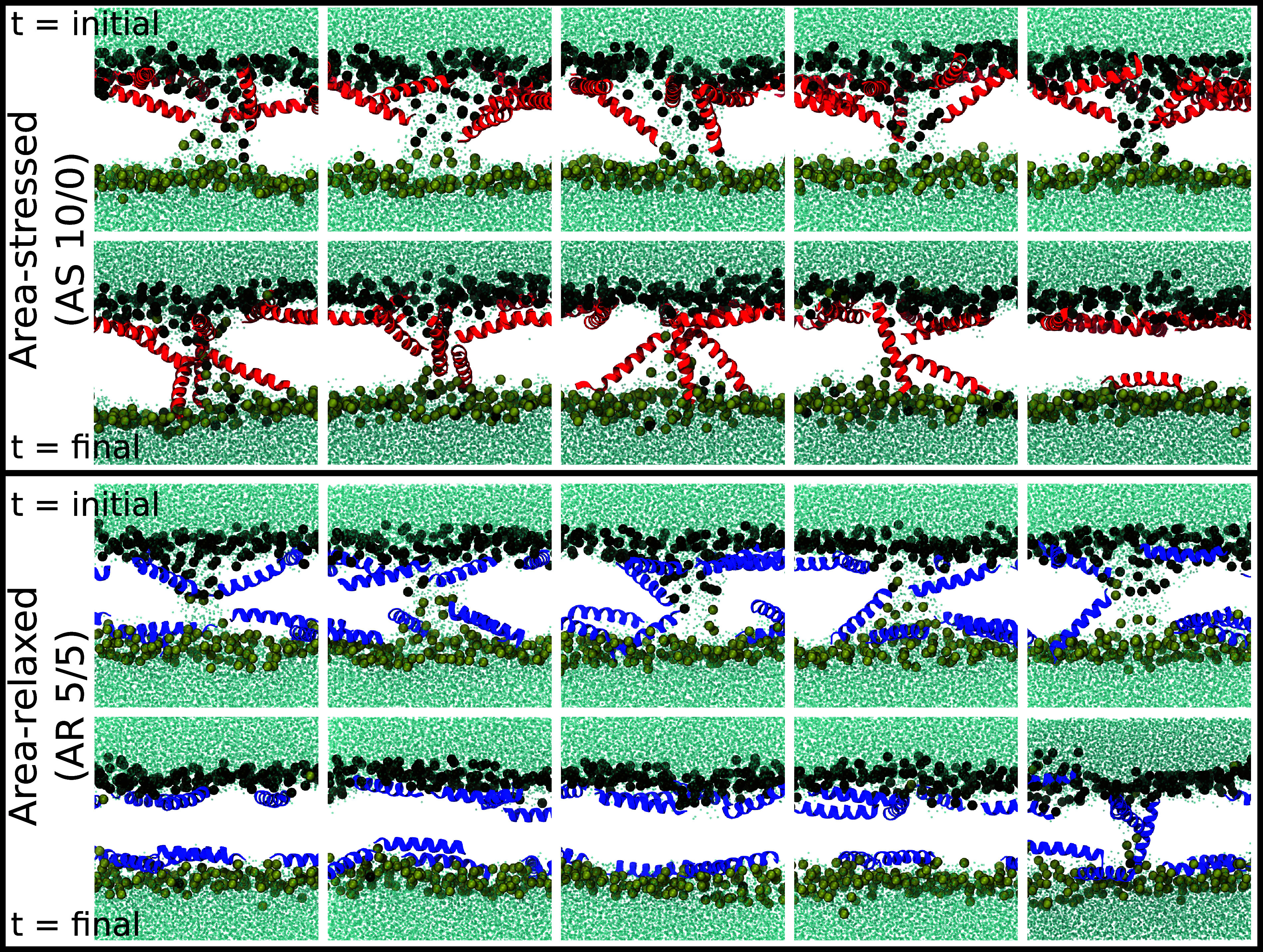
Initial and final snapshots from unbiased simulations of a 10.5 Å radius pore (ξ=2.48) in AS 10/0 and AR 5/5. Simulation lengths were 3 µs for AS 10/0 systems (*Top* panels), 1 µs for AR 5/5 (*Bottom* panels) replicates 1 to 4, and 3 µs for AR 5/5 replicate 5. Water is depicted as cyan dots, peptides are red or blue helical ribbons, and lipid phosphorus atoms are spheres colored according to which leaflet they began in when the systems were constructed—black for top leaflet and gold for bottom leaflet. Note the presence of multiple black (top leaflet) headgroups in the bottom leaflet for the final snapshots of AS 10/0 systems.

To assess pore stability and the translocation of lipids and peptides through the pore, 10 unbiased pore simulations were performed each for the AS 10/0 and AR 5/5 systems starting from CPP-like (ξ=1.18, r=5.0 Å) or fully hydrated pores (ξ=2.48, r=10.5 Å). Fig. 5 and *SI Appendix*, Fig. S7 show initial and final snapshots of each replicate of the fully hydrated and CPP-like pores, respectively.

In the simulations of fully hydrated pores (ξ=2.48), four of the five fully hydrated pores in the AS 10/0 systems were stable for the entire 3 µs simulations, with the pore structures evolving over the trajectories. In some replicates, peptides translocated through the pore to stabilize it from the opposite leaflet. Additionally, there was a marked and rapid translocation of lipids through the pore, with an average of 10.8 ± 0.4 net lipid translocations from the top leaflet to the bottom leaflet (*SI Appendix*, Table S4). Lipid translocation occurred very quickly in these systems, with about half (5.6 ± 1.2) of the translocation events occurring during the 100 ns pore opening simulations, prior to either the US simulations or unbiased pore simulations. The remaining lipid translocations were mostly completed within the first ∼250 ns of the unbiased simulations, with the lipid leaflet distributions remaining roughly stable beyond that point (*SI Appendix*, Fig. S8). In the fifth replicate, the pore spontaneously closed after 2.3 µs, with one peptide translocating to the opposite leaflet. In contrast, four of the five pores in the AR 5/5 simulations closed within the first 120 ns of the simulation (t = 28, 92, 112, 113 ns). For these four replicates, essentially no lipid translocation occurred before the pores closed (*SI Appendix*, Fig. S8 and Table S4). In the fifth replicate, the pore remained intact for the full 3 µs trajectory, though for much of the trajectory the structure was that of a single transmembrane peptide with few associated water molecules in the hydrophobic portion of the bilayer and no associated lipid head groups.

For simulations of CPP-like pores, three of the five AS 10/0 pores dissipate within 65 ns (t = 20, 44, 63 ns) while the remaining two pores stayed open for the full 1 µs-long trajectories (*SI Appendix*, Fig. S7). In these two replicates, the pores grew during the simulation, becoming more hydrated and expanding to sizes similar to those of the 10.5 Å pore simulations. These results are consistent with both the small local maximum in the AS 10/0 free energy profile at ξ≈1.1 as well as the broad, shallow minimum for ξ=1.11 to 1.83. There were an average of 1.33 ± 0.40 lipid translocations in the replicates where the pores closed, and 11.92 ± 1.77 translocations in the two replicates where the pores grew in size. In the AR 5/5 replicates, all five CPP-like pores dissipated within the first 40 ns of the simulation (t = 6, 17, 18, 36, 37 ns) and there was no net lipid translocation.

### Peptide Distribution Is Expected to Be Inhomogeneous on an LUV Surface.

The simulations presented here assume peptides are evenly distributed across the surface of the vesicle (the primary simulation cell and all periodic image cells are identical); however, variability is expected in the local distribution of peptides on the vesicle surface. As an example, consider a 100 nm diameter LUV which, assuming 65 Å^2^ as the average area per lipid (49), contains 100,000 lipids and a leaflet surface area of 32,500 nm^2^, or approximately 325 patches of the size used in the simulations (10 × 10 nm^2^). At L/P = 30, the LUV would contain 3,333 peptides, or an average 10.3 peptides in each patch. However, the peptides would not be expected to be equally distributed across the membrane, even if they are assumed to be noninteracting.

**Fig. 6.**
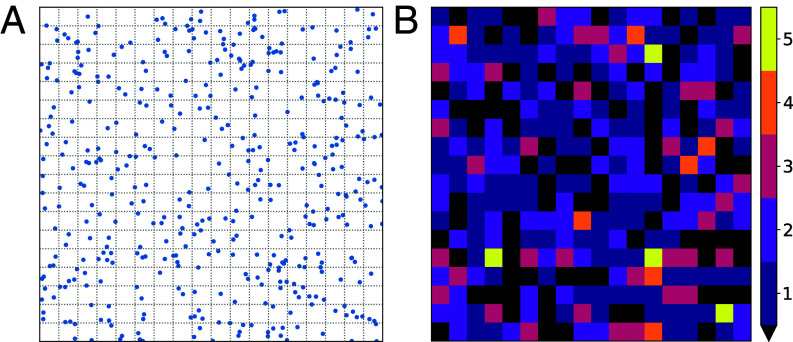
Peptide inhomogeneity on an LUV surface. (*A*) Points (representing peptides) randomly distributed on an 18×18 grid modeling a 100 nm diameter vesicle subdivided into 100×100 Å grids at a L/P of 238. (*B*) Same L/P = 238 distribution represented as a heat map of peptides per bin. Black squares are bins with no peptides.

*SI Appendix*, Table S5 lists the means and variances for the expected number of noninteracting peptides per 10×10 nm^2^ patch when randomly distributed on a surface area consistent with a 100 nm diameter LUV for L/P = 30, 60, 228, and 478. The first two ratios were used for MD simulations in this paper, and all four are within the range measured experimentally. Fig. 6 depicts points (symbolizing peptides) randomly distributed on a surface area consistent with a 100 nm diameter LUV for L/P = 228, and *SI Appendix*, Fig. S9 shows two additional L/P ratios. The variance is close to the mean in all cases, and even at the higher peptide concentrations, the probability of having regions with only one or two peptides is nonnegligible. Furthermore, if interactions between peptides in the membrane are attractive, the heterogeneity of peptide distribution will be even more pronounced.

### Unified Leakage Model Reproduces All-or-None, Graded, and Mixed Behavior.

All-or-none and graded release have two different sets of underlying assumptions for how the probability of a vesicle leaking ($P_{\mathrm{leak}}^{v}$) and the extent of its leakage ($f_{\mathrm{out}}^{v}$) vary with peptide concentration ($c_{p}$). All-or-none release assumes that vesicles either leak completely or do not leak at all and that the probability of any given vesicle leaking is related to the concentration of peptide present; in other words, $f_{\mathrm{out}}^{v}=0$ or 1, and $P_{\mathrm{leak}}^{v}=f$($c_{p}$). Graded release assumes the opposite, that vesicles always leak and that the extent of a vesicle’s leakage ($f_{\mathrm{out}}^{v}$) is a function of peptide concentration; in other words, $P_{\mathrm{leak}}^{v}=1$ and $f_{\mathrm{out}}^{v}=f$($c_{p}$). The model presented here addresses the case where both $P_{\mathrm{leak}}^{v}$ and $f_{\mathrm{out}}^{v}$ are allowed to vary with peptide concentration.

**Fig. 7.**
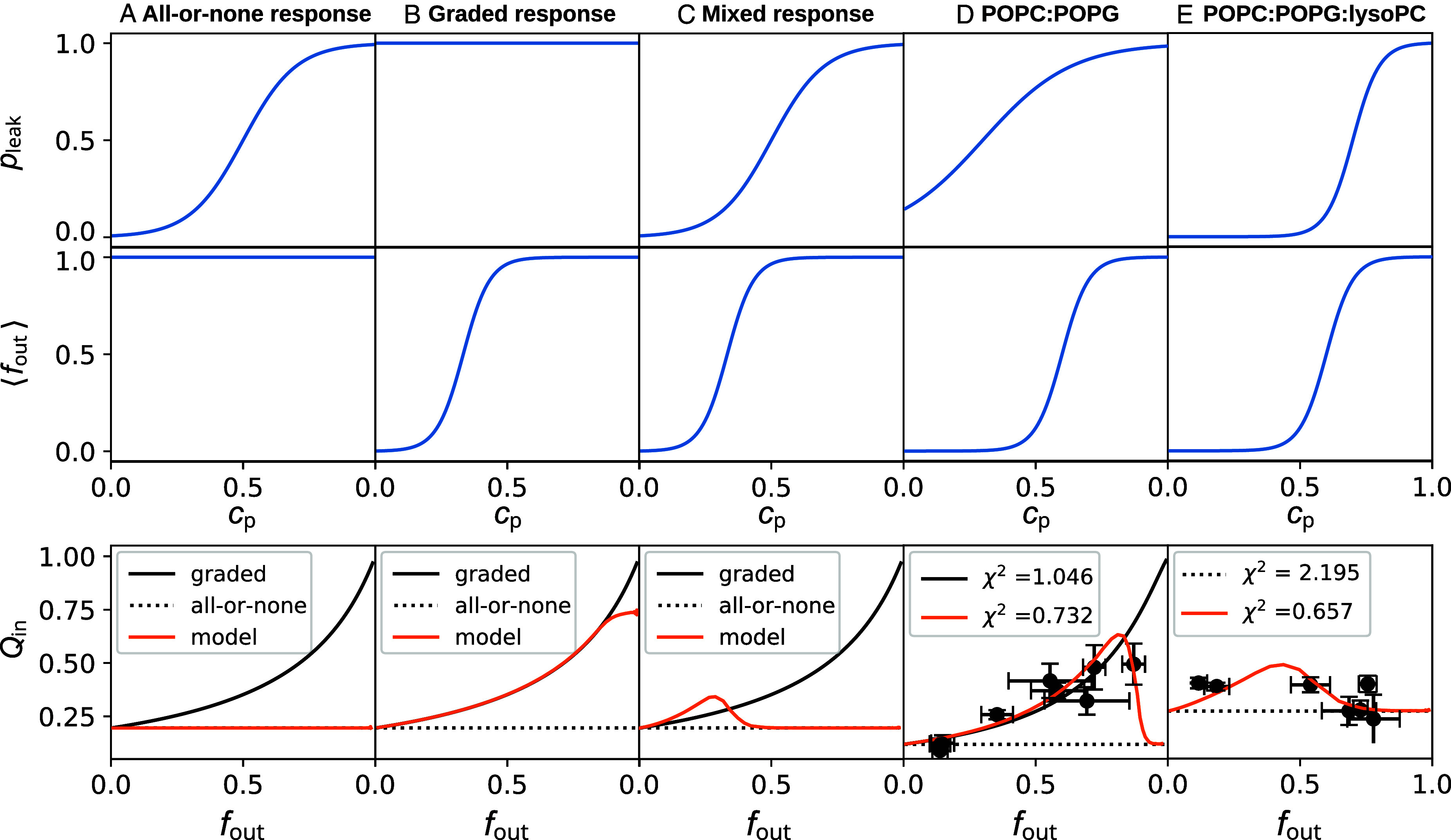
Prediction from the unified model that recapitulates the molecular interactions underlying the transient leakage induced by AMPs. Model output predicts (*A*) ideal all-or-none leakage, (*B*) ideal graded leakage, or (*C*) a nonmonotonic mixed leakage behavior depending on how the probability of leakage $P_{\mathrm{leak}}$ and the fractional vesicle leakage $\langle f_{\mathrm{out}}\rangle$ vary with peptide concentration $c_{p}$. Model fit to experimental data for P1 in (*D*) 3:1 POPC:POPG and (*E*) 2:1:1 POPC:POPG:lysoPC. See text for parameters used for *D* and *E*. For both lipid systems, the unified model yields a better goodness-of-fit $\chi^{2}$ to the experimental data than does ideal graded or all-or-none, though it utilizes four adjustable parameters instead of two. For all, top two plots show $P_{\mathrm{leak}}$ and $\langle f_{\mathrm{out}}\rangle$, while the corresponding bottom plots demonstrate the model output under the circumstances specified.

Fig. 7 shows that the modeled data accurately reproduces the idealized all-or-none and graded behavior and can be fit to experimental data as well. When $\langle f_{\mathrm{out}}\rangle$ = 1 the modeled data demonstrate the flat line behavior of all-or-none leakage (Fig. 7*A*), and when $P_{\mathrm{leak}}=1$ the model output matches the graded response curve (Fig. 7*B*). However, when both $P_{\mathrm{leak}}$ and $\langle f_{\mathrm{out}}\rangle$ are allowed to vary with peptide concentration, the model predicts a nonmonotonic “mixed” response, with graded-like behavior at lower peptide concentrations and all-or-none behavior at higher peptide concentrations (Fig. 7*C*). The predicted nonmonotonic behavior of the model, with a decreasing or mostly flat response at larger $f_{\mathrm{out}}$ coupled with a small rise at lower $f_{\mathrm{out}}$ can be seen in published data for many AMPs that are reported to permeabilize in an all-or-none fashion, including magainin 2 (15), cecropin A (14) and its mutant (50), as well as some designed α-helical peptides (20). The location of the turnover point, from graded to all-or-none behavior, and the steepness of the curve can be varied depending on the specifics of how both $P_{\mathrm{leak}}^{v}$ and $f_{\mathrm{out}}^{v}$ vary with $c_{p}$ (*SI Appendix*, Fig. S10), as well as the value of α assumed.

As demonstrated in Fig. 7 *D* and *E*, this single model provides excellent fits to the experimental data collected for P1 with both lipid compositions studied here (Fig. 2 *E* and *F*). For POPC:POPG, the fit parameters are $C_{1}=3$ and $C_{2}=0.4$ for $P_{\mathrm{leak}}\left(c_{p}\right)$, and $C_{1}=10$ and $C_{2}=0.7$ for $\langle f_{\mathrm{out}}\left(c_{p}\right)\rangle$, where the $C_{1}$ parameters affect the steepness of the sigmoid functions and the $C_{2}$ parameters control the midpoint. The unified model provides a better fit to the experimental data than the ideal graded model of *SI Appendix*, Eq. **S8**, improving $\chi^{2}$ from 1.046 to 0.732. However, the unified model uses four adjustable parameters instead of the two adjustable parameters present in *SI Appendix*, Eq. **S8**. For POPC:POPG:lysoPC, the fit parameters used are $C_{1}=10$ and $C_{2}=0.7$ for $P_{\mathrm{leak}}\left(c_{p}\right)$, and $C_{1}=10$ and $C_{2}=0.6$ for $\langle f_{\mathrm{out}}\left(c_{p}\right)\rangle$. The unified model improves the goodness of fit with $\chi^{2}=0.859$, compared to 2.195 for ideal all-or-none with [DPX]_0_ = 0.004 M or 1.805 with [DPX]_0_ = 0.003 M.

## Discussion

In this work, experimental dye leakage results demonstrate that poration by P1 is transient, with graded release in 3:1 POPC:POPG and predominantly all-or-none in 2:1:1 POPC:POPG:lysoPC (Fig. 2). In both cases, leakage begins quickly after peptide addition, stops after some time, and can be reinduced by addition of more peptide. The larger number of defects previously observed by MD in POPC:POPG:lysoPC compared to POPC:POPG in Paper 1 correlate with the faster dye efflux rate, greater peptide potency, and all-or-none mode of leakage in the lysoPC-containing LUVs presented here. In contrast, the fewer number of defects and slower efflux rate observed in POPC:POPG are associated with graded leakage and a weaker peptide potency. This result is consequential since it identifies molecular aspects of the membrane (e.g., packing defects, positive spontaneous curvature) as properties that influence not only the leakage mode (graded vs. all-or-none) but also its kinetics (rate of dye release) and potency (EC_50_ value).

To model the experimental system in its initial and final conformations, lipid bilayers were simulated at three different L/P ratios in 2:1:1 POPC:POPG:lysoPC with peptides either symmetrically or asymmetrically distributed. Bilayer thickness fluctuations and defect formation were both greatly enhanced in the asymmetric AS systems compared to the symmetric AR systems, consistent with the notion that area stress generated by peptide asymmetry induces pore formation (*SI Appendix*, section S1.1). Furthermore, the differences in defect rates and leaflet fluctuations between AS/AR systems correlated well to the degree of area mismatch in the systems (*SI Appendix*, Table S2).

**Fig. 8.**
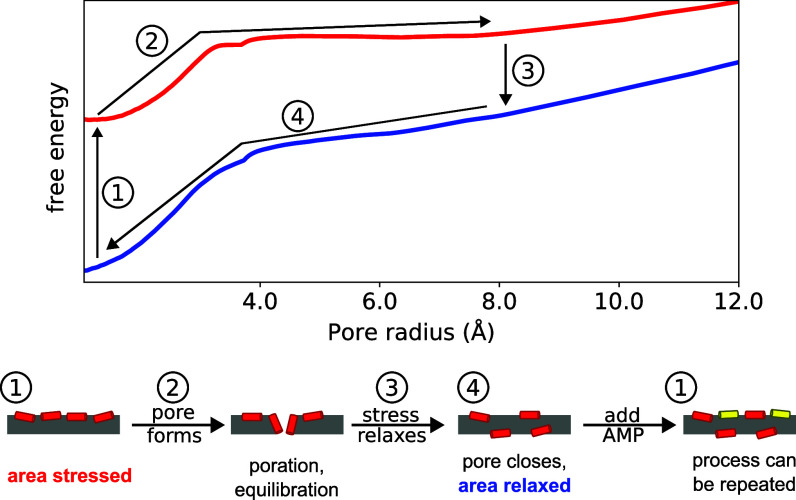
Mechanism of peptide-induced transient leakage, with energy landscape. Leakage follows the process of (1) peptides bind to a relatively unstressed membrane and induce area stress (2) metastable pores form (3) lipids and peptides translocate through the pore, area stress is reduced, and (4) pores close. Whether the observed leakage exhibits all-or-none or graded characteristic depends on the kinetics of pore formation, pore dissipation, and dye leakage. The AS 10/0 (red) and AR 5/5 (blue) free energy profiles have been shifted relative to one another to indicate the energetic penalty associated with area stress (step 1) and the spontaneous nature of asymmetry relaxation through the pore (step 3).

The free energy of pore formation was directly calculated in 2:1:1 POPC:POPG:lysoPC and was significantly higher for AR peptide distributions than AS ones (Fig. 3), directly demonstrating the effects of asymmetry and area stress on the free energy landscape of pore formation. Area stress significantly decreases the energy barrier to pore formation, by as much as 10.3 kcal/mol at L/P = 30. One reason for the observed difference in pore energy and stability is that in the AS systems peptides inserted more deeply as the pore formed, and more peptides were recruited to the pore as it grew (Fig. 4). The shape of the energy landscape is also different, permitting formation of a metastable pore under AS conditions but not AR ones. Simulations of the formed pores demonstrated rapid translocation of lipids and occasionally peptides through the pore, consistent with earlier experiments and simulations (51–53). From these results, a rigorously determined energetic description can be added to the mechanistic cartoon of transient leakage, validating the long-accepted mechanism of transient leakage as driven by asymmetry stress (Fig. 8). Vesicles are initially in an equilibrium state prior to the addition of peptide. When the peptide is introduced to the system, it is initially bound only to the outer leaflet of the vesicle leading to a mismatch in surface area and a resulting differential stress. As seen in the calculated energy landscape, this stress greatly decreases the energy needed to form a pore and favors the formation of metastable pores. Lipids and peptides are able to translocate through the metastable pore, re-equilibrating the lipid and peptide distributions and relaxing the area stress within the system. As the stress is relaxed, metastable pores are no longer energetically favorable and the pores spontaneously close. Introducing additional peptide reestablishes asymmetry, resulting in area stress and additional poration which subsequently subsides.

The requenching assays performed indicate that P1 changes from graded leakage behavior in 3:1 POPC:POPG LUVs to predominantly all-or-none in 2:1:1 POPC:POPG:lysoPC LUVs (Fig. 2). The poration free energy simulations performed in these two lipid compositions demonstrate that the mechanism of pore formation and closure is similar in both; the primary differences are that it takes less energy to form a pore in 2:1:1 POPC:POPG:lysoPC and the pores that form are larger than in 3:1 POPC:POPG (Fig. 3). While this is the first demonstrated example of a transition from graded to all-or-none with lipid composition, several studies have shown that vesicle size affects whether or not leakage is classified as graded or all-or-none (31, 32, 54, 55), indicating that the two exist along a spectrum of leakage behaviors. The present results are consistent with this interpretation.

The unified model presented in Fig. 7 allows computation of the expected fluorescence requenching results for a spectrum of graded/all-or-none leakage behaviors, where both the likelihood a vesicle leaking and the extent of its leakage are influenced by peptide concentration, combining the assumptions of idealized graded and all-or-none leakage. At lower peptide concentrations (i.e., lower $f_{\mathrm{out}}$), there is a rise in $Q_{\mathrm{in}}$ corresponding to a graded-like leakage behavior that becomes more all-or-none as $Q_{\mathrm{in}}$ flattens out or decreases at higher peptide concentrations. The location of the inflection point from graded-like to all-or-none behavior depends on the specifics of how both the leakage probability $p_{\mathrm{leak}}$ and the average fractional release $\langle f_{\mathrm{out}}\rangle$ vary with peptide concentration (*SI Appendix*, Fig. S10). This nonmonotonic response of $Q_{\mathrm{in}}$ with increasing $f_{\mathrm{out}}$ is present in the experimental requenching data for P1 in 2:1:1 POPC:POPG:lysoPC (Fig. 2) as well as magainin 2 (15), cecropin A and one of its mutants (14, 50), and some designed α-helical peptides (20). To our knowledge, this is the first leakage model to predict a nonmonotonic “mixed” response of $Q_{\mathrm{in}}$ with increasing $f_{\mathrm{out}}$. The nonmonotonicity not only enables a better fit to experimental requenching data than other models but also substantiates the hypothesis of a single mechanism for the full spectrum of leakage behaviors.

**Fig. 9.**
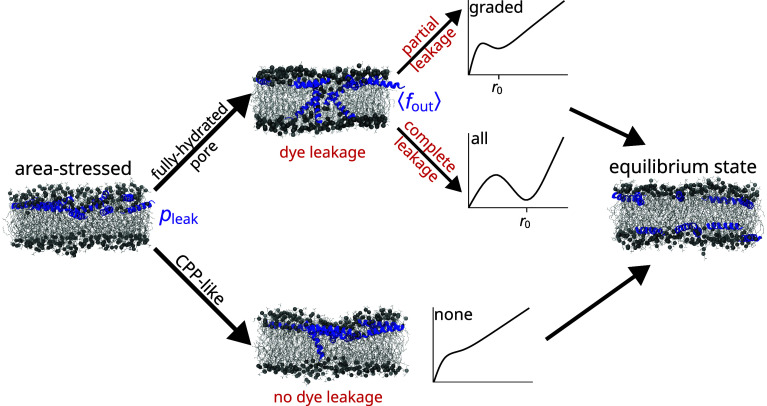
Molecular model of peptide-induced transient membrane leakage. Peptide-induced area stress can be relieved by formation of either a CPP-like intermediate, where lipids and peptides translocate without appreciable release of vesicle contents, or a fully hydrated metastable pore, which allows dye release; both processes are always present, and competition between them is described by the probability of vesicle leakage $P_{\mathrm{leak}}$ in our model. Fully hydrated pores with small radii that close quickly will only partially leak vesicle contents leading to graded behavior, while pores that are larger and relatively long-lived can release most of the vesicle contents resulting in all-or-none behavior. In our model, the spectrum between these two possibilities is described by $\langle f_{\mathrm{out}}\rangle$, the average fractional leakage from vesicles. Both the CPP-like and fully hydrated pore pathways result in an equilibrium state where transbilayer area stress has been reduced. The included graphs are schematic diagrams of free energy as a function of pore radius, with the metastable radius denoted by $r_{0}$.

Furthermore, the rigorous simulations and quantitative modeling performed in this work provide an explanation for varying poration strength and likelihood within the framework of asymmetry-driven poration. The simulations demonstrate that while complete, partial, or an absence of vesicle leakage are all driven by the same asymmetry stress mechanism, the primary differences arise from the relative size and lifetimes of the pores formed. Fig. 9 sketches the simulation-based mechanism, motivated in part by that of Rathinakumar and Wimley (20). When peptides are first added to the vesicles, their presence on the outer leaflet induces significant asymmetry and area stress. There are two pathways to relieving this stress through pore formation: a CPP-like pathway where lipids and peptides are able to translocate without appreciable dye release (represented by the 5 Å pores in *SI Appendix*, Fig. S7) and a fully hydrated pore pathway involving formation of a metastable disordered toroidal pore large enough to release dye (the 10.5 Å pores in Fig. 5). The basis of these two pathways is the nonuniform distribution of peptides across the vesicle (Fig. 6); regions of low peptide abundance will favor the CPP-like pathway while regions of high peptide abundance will favor the fully hydrated metastable pore pathway. Both pathways are expected to occur simultaneously and in competition with one another; a consequence of this competition is that the probability of observing dye release decreases with time (20, 24). The relative likelihood of these two pathways is the physical interpretation of $P_{\mathrm{leak}}$ in our model, and depends on the peptide concentration (as seen in the poration free energy profiles of Fig. 3 calculated at different L/P) as well as the lipid composition of the membrane.

Within the fully hydrated pore pathway, the extent of dye release from a vesicle depends on the kinetics of pore formation, pore closure, and the difference in peptide concentration between outer and inner leaflets. If the formed metastable pores have a small radius and low energetic barrier to closing, they will close before much or all of the vesicle’s dye is released, leading to graded-like behavior. Metastable pores with larger radii and higher energetic barriers to closing will allow more dye to be released before closing, contributing to an all-or-none-like behavior (32). Faster dye flux has been shown to correlate with complete (all-or-none) release (56, 57), an observation further demonstrated in this study with P1 (Fig. 2), again confirming that larger, longer-lived pores lead to all-or-none release.

As part of this framework, peptides that are strong pore formers and/or are acting on membrane compositions that can be more easily porated will induce all-or-none leakage. At low peptide concentrations, these peptides will still be highly active through the CPP-like pathway, relieving area stress without appreciable dye leakage. However, in some cases, the local peptide concentration will be much higher than average (Fig. 6) allowing the formation of larger, fully hydrated pores that leak dye quickly. At higher peptide concentrations, these large pores will form faster and with a higher probability, though competition will still exist with the CPP-like pathway. This leads to an all-or-none or all-or-some leakage behavior where some vesicles do not leak but those that do leak nearly completely. On the other hand, peptides that are weaker pore formers and/or are acting on membrane compositions that are more difficult to porate induce graded leakage since both the CPP-like and fully hydrated pore pathways are less energetically favored, and the metastable pores that form will close before much dye can leak. This results in a graded or some-or-none leakage behavior where most of the vesicles leak incompletely.

To predict or modify graded, all-or-none, and intermediate leakage behavior, the lipid composition of the membrane, the properties of the specific peptide, the local peptide concentration, and the extent of area stress must be considered since they all contribute to the poration free energy landscape and determine the leakage behavior. For P1, the basis of the change from graded in 3:1 POPC:POPG to all-or-none in 2:1:1 POPC:POPG:lysoPC is that lysolipids induce positive monolayer spontaneous curvature; this increases both the size of the pore and the energy barrier to pore closure (48), an effect illustrated by the poration free energies calculated for these lipid compositions (Fig. 3). If interactions between peptides in the membrane are attractive, the surface heterogeneity will be more pronounced, promoting formation of fully hydrated pores in regions where the local concentration is high and CPP-like pores in regions where the local concentration is low; thus heterogeneity or a propensity to cluster on the membrane surface, especially where defects are present, will favor all-or-none leakage, while homogeneity of peptide distribution will favor graded leakage behavior (31). Finally, the specifics of the peptide, such as its sequence, hydrophobicity, and ability to partition into the membrane will affect the energy needed to form a pore, as well as its stability and size.

Peptide-specific effects could be examined in more detail by calculating poration free energies across multiple AMPs and CPPs, ultimately allowing for accurate predictions of leakage mode and interaction strength for different peptide-lipid systems as well as rational design of peptides that can selectively distinguish and disrupt targeted membranes. While this study focused on the mechanisms underlying AMP-induced transient membrane poration, the conclusions drawn are applicable to many classes of MAPs. Furthermore, the ability to predict for a given amino acid sequence and lipid composition the surface heterogeneity of the peptides and the defect propensity of the membrane could be leveraged to design peptides that have enhanced specificity for cells of interest based on their plasma membrane compositions.

## Conclusions

First, the dye leakage experiments demonstrate that the same peptide (piscidin 1) can produce graded or all-or-none leakage, depending only on the lipid composition of the vesicles. Results from MD simulations show that this difference is due to variation in the barriers to pore formation and the stability of larger pores, validating the notion that large, more stable pores lead to all-or-none leakage while small, less stable pores promote graded leakage (32). It was also shown that pores too small to leak dye still generate lipid translocation; they are the “none” component of the all-or-none leakage, and bear a resemblance to cell-penetrating peptides. The MD simulations enabled visualization of the pore structures responsible for these leakage behaviors, depicting not only the transbilayer movement of lipids but also peptides as part of the transient process associated with relieving asymmetry stress. Second, the free energy calculations of pore formation provide quantitative measurements of the effects of area stress and asymmetry on poration energy, supporting the long-accepted mechanism of transient leakage as driven by asymmetry stress (4, 18, 22–24). Third, the quantitative model of fluorescence requenching is based on the idea that graded and all-or-none leakage are two extremes along a continuum (23, 30, 31), and allows for modeling of mixed leakage behavior. This model predicts nonmonotonic behavior in the requenching response curves, an effect seen in our experimental data for P1 as well as many that have been published for other AMPs (14, 15, 20, 50). The improved fit of this model to the experimental data bolsters the continuum view of leakage behavior and allows an estimate of the relative sizes of membrane defects.

Our unified mechanistic model integrates transient leakage and key molecular aspects, including the lipid composition, local peptide concentration, and extent of area stress. This approach could be consequential for the nascent field of MAP design, which has largely focused on mutating existing sequences to improve function and selectivity (1–8, 58). Mechanistic insights are typically achieved retroactively, yielding conclusions valid for a given sequence and experimental conditions, but with limited generalizability; this lack of predictive power is a bottleneck that the field must overcome to achieve broad translational impact. Our work represents a step towards development of a bottom–up approach to MAP design. Integrating the unified model with machine learning (ML) (59–63) could enable the in-silico evolution of novel sequences based on learned patterns. For AMPs in particular, combining requenching data with antimicrobial potency and cytotoxicity measurements would allow for a broader exploration of the sequence-activity-mechanism landscape to help identify which peptide sequences, membrane compositions, and leakage mechanism (e.g., graded vs. all-or-none) correlate with the greatest selectivity and most effective antimicrobial activity.

## Materials and Methods

### Materials.

LysoPC was selected rather than lysoPG because the former has been examined in numerous studies of curvature strain (64, 65), including our own on P1 (39) and the influenza A fusion peptide (66). POPC, POPG, and 16:0 lysoPC were purchased from Avanti Polar lipids (Alabaster, AL) and Anatrace Inc. (Maumee, OH). 1,2-dioleoyl-sn-glycero-3-phosphoethanolamine-N-[amino(poly-ethylene glycol)-2000], ammonium salt (PEG-2k) was a gift from the Wimley lab (Tulane University), who purchased it from Avanti Polar Lipids. The fluorescent dye 8-aminonaphthalene-1,3,6-trisulfonic acid, disodium salt (ANTS) and the quencher p-xyleen-bis-pyridinium bromide (DPX) were purchased from Invitrogen (Waltham, MA). The self-quenching calcein dye was purchased from Millipore Sigma (Burlington, MA). All other reagents were purchased from Fisher Scientific (Hampton, NH).

### Peptides.

Carboxy-amidated piscidin 1 (sequence: FFHHIFRGIVHVGKTIHRLVTG-NH_2_) was made by solid phase peptide synthesis at the Tufts University Core Facility (Boston, MA), with purification performed as previously reported by Perrin et al. (67). See *SI Appendix*, section S2.1 for further details.

### Preparation of Large Unilamellar Vesicles.

The requenching assay followed the procedure published by Wimley et al. (27–29) with minor modifications (*SI Appendix*, section S2.2). The start-stop experiment used calcein-loaded vesicles, as previously described (45) with an 80 mM calcein solution used during the hydration step.

### Start–Stop Dye Leakage Assay.

For each lipid system, calcein-loaded LUVs of each lipid system were plated in triplicate then treated with peptide at L/P = 478, 239, 119, or 60. The fluorescence was monitored until it stabilized (∼2 h). Then, a second P1 addition was made using the same amount of peptide as in the initial addition and the fluorescence was monitored again until it reached another plateau (∼1.5 h). The same assay was also performed with ANTS/DPX, confirming the same trends. For each time point that a measurement was made, the fractional leakage was calculated in triplicate, then plotted with their SD as error bars. To estimate the rates of the fluorescence bursts at each L/P, a single well measurement was performed in duplicate, and first derivatives of the fluorescence were calculated from the first 10 s of readings. Further details are in *SI Appendix*, section S2.3.

### High-Throughput Fluorescence Requenching Assay.

ANTS/DPX loaded LUVs were used for the requenching assay. The protocol for the leakage assay developed by White et al. (28, 29) was adapted to a 96-well plate high-throughput setup where each condition was plated in triplicate. A full description of the experimental setup and data analysis are in *SI Appendix*, section S2.4.

### Calculation of EC_50_ Values.

From the fluorescence measurements made for the requenching assay, the fractional leakage after 1 h incubation induced at all L/P ratios was used to build a dose–response curve (x = fractional leakage vs. y = L/P ratio). The normalized y vs. x data was fitted in GraphPad Prism (GraphPad, San Diego, CA) to yield the half-maximal effective L/P (EC_50_) of the peptide via the following equation:

[1] $$\begin{array}{c} y=1-\frac{1}{1+{\left(\frac{x}{{\mathrm{EC}}_{50}}\right)}^{p}}, \end{array}$$

where p is a cooperativity coefficient.

### MD System Preparation.

All systems were constructed using CHARMM-GUI (68) with 150 lipids of composition 2:1:1 POPC:POPG:16:0 lysoPC in each leaflet (300 lipids total) and 150 mM sodium chloride in the solvent. Multiple copies of P1 in α-helical conformations were placed in either the top leaflet or both leaflets, with the peptides evenly spaced and at random orientations with respect to one another. Six systems were constructed: 5 peptides in the top leaflet (AS 5/0), 3 peptides in each leaflet (AR 3/3), 10 peptides in the top leaflet (AS 10/0), 5 peptides in each leaflet (AR 5/5), 10 peptides in the top leaflet and 5 in the bottom (AS 10/5), or 7 peptides in each leaflet (AR 7/7). The P1 C termini were amidated to match the experiments, and all histidine residues were modeled in the neutral state for consistency with the measured p$K_{a}$ values (45). Simulations utilized the CHARMM 36 force field (69, 70) and TIP3P water (71, 72).

### Conventional MD Simulations.

Conventional MD simulations were run with OpenMM version *7.4.1* (73) and Anton 2 software version *1.57.1c7* (74), and were performed in the isothermal–isobaric ensemble (T = 310 K and P = 1 atm). Additional simulation details are given in *SI Appendix*, section S2.5.

### Pore Opening and US Simulations.

Eight PMFs were calculated, all six systems in this work—AS 10/0, AR 5/5, AS 10/5, AR 7/7, AS 5/0, and AR 3/3—and two systems from Paper 1: AS 10/0 in 3:1 POPC:POPG and a 10/0 peptide distribution in 2:1:1 POPC:POPG:lysoPC (denoted as AR 10/0) where bilayer stresses have been relaxed through P2_1_ boundary conditions (75). US calculations used the extended pore reaction coordinate ξ (76, 77), implemented via a custom version of GROMACS 2021.2. Values of ξ≲1 quantify pore nucleation with the chain coordinate (78), while values of ξ>1 quantify pore expansion by the pore radius relative to a reference radius R_0_, which corresponds to the radius when ξ≈1. Initial pore opening simulations were performed to seed the US windows; five replicates were generated for each system, starting from defect frames taken from the unbiased simulations. Coordinates from each of these replicates were used as initial coordinates for the US windows. For each PMF, 36 windows were simulated ranging from ξ=0.065 to 2.93. Each window was simulated five times, with each replicate starting from one of the five pore opening simulations, and a simulation length of 225 ns per replicate; the first 25 ns were removed as equilibration. This yielded 1 µs of sampling per window, and 36 µs in aggregate per PMF. See *SI Appendix*, section S2.6 for further details.

### Unbiased Pore Simulations.

To assess pore stability, as well as lipid and peptide translocation through the pore, unbiased pore simulations were performed for AS 10/0 and AR 5/5. For both systems, snapshots at ξ=1.18 (r = 5.0 Å, CPP-like pores) and ξ=2.5 (r = 10.5 Å, fully hydrated pores) were taken from each of the five pore opening simulation replicates (two from each replicate). These pores were then simulated without any biasing potential for up to 3 µs each in OpenMM, using the simulation parameters described in *SI Appendix*, section S2.5. In simulations where the pore closed, the simulation was terminated shortly after pore closure.

### MD Simulation Analysis.

Percent area mismatch ϕ was defined as in ref. 33 to be the difference in equilibrium area between the top and bottom leaflets, relative to the top (larger) leaflet. Specifics of this calculation are given in *SI Appendix*, section 2.7. Leaflet surface maps and bilayer thickness maps were calculated using the MEMBPLUGIN (79) tool in VMD (80), and defects were identified using the protocol introduced in Paper 1 (39). In pore simulations, lipid and peptide translocations were assessed by tagging peptides and lipid headgroups based on which leaflet they were originally placed in when the systems were constructed, and monitoring their positions over the course of the simulations (details available in *SI Appendix*, section S2.7). The weighted histogram analysis method (WHAM) implemented in GROMACS (81) was used to analyze US simulations and compute PMFs, with the error calculated through a Monte Carlo bootstrapping error analysis. System snapshots were rendered using VMD (80).

### Requenching Assay Modeling.

In requenching experiments, the measurement is an average over all vesicles in the sample and the internal fluorescence $Q_{\mathrm{in}}$ and fractional leakage $f_{\mathrm{out}}$ are interpreted as bulk quantities. However, the same relation (*SI Appendix*, section S2.8 and Eq. **S8**) can be applied to a single vesicle, where $Q_{\mathrm{in}}^{v}$ and $f_{\mathrm{out}}^{v}$ denote the internal quenching and fractional leakage of a single vesicle, as opposed to the bulk properties. In this model, $Q_{\mathrm{in}}^{v}$ and $f_{\mathrm{out}}^{v}$ for a single vesicle are calculated through a two step process, where the likelihood of leaking, $P_{\mathrm{leak}}$, and the average extent of leaking, $\langle f_{\mathrm{out}}\rangle$, are separate and allowed to vary as a function of peptide concentration $c_{p}$, where $c_{p}$ is expressed in arbitrary units ranging from 0 to 1. $P_{\mathrm{leak}}($c_p_) and $\langle f_{\mathrm{out}}\left(c_{p}\right)\rangle$ are represented by sigmoid functions of $c_{p}$, or expressed as constant values in the range [0,1] in keeping with the assumptions of all-or-none or graded leakage. Additional details are given in *SI Appendix*, section S2.8.

## Supplementary Material

Appendix 01 (PDF)

## Data Availability

Experimental fluorescence data and a Python script for the unified leakage model of fluorescence requenching have been deposited in Zenodo (https://doi.org/10.5281/zenodo.16542724) (82). Some study data are available: all MD trajectories generated on Anton 2 can be accessed by emailing the Pittsburgh Supercomputer Center (PSC) at anton-support@psc.edu. The remaining MD trajectories are available on the LoBoS cluster at NIH (contact R.W.P. at pastorr@nhlbi.nih.gov).
