# Supplementary material for: A unified model of transient poration induced by antimicrobial peptides
Source: Proc Natl Acad Sci U S A. 2025 Aug 29;122(35):e2510294122. doi: 10.1073/pnas.2510294122 (PMC12415194; doi:10.1073/pnas.2510294122)
Supplement: Supplementary file 1 — Appendix 01 (PDF) [file pnas.2510294122.sapp.pdf]

Supporting Information for

A unified model of transient poration induced by antimicrobial peptides

Amy Rice, Andriana C. Zourou, Myriam L. Cotten, Richard W. Pastor

Myriam L. Cotten, E-mail: cottenmy@oregonstate.edu
Richard W. Pastor, E-mail: pastorr@nhlbi.nih.gov

This PDF file includes:

Supporting text
Figs. S1 to S10
Tables S1 to S7
SI References

Supporting Information Text

S1 Supplementary Results

S1.1 Area stress enhances defect formation. Consistent with Paper 1 (1), for all six systems of P1 in 2:1:1 POPC:POPG:lysoPC simulated, the presence of P1 affects bilayer structure through a modest thinning of the hydrophobic thickness, greater lateral thickness fluctuations, and larger undulations in the leaflet surface (Table S2). The effect of area stress on the average hydrophobic thickness is small, leading to less than 1 Å of thinning even when comparing area-stressed (AS) 10/0 (24.95 ± 0.01 Å) and area-relaxed (AR) 5/5 (25.60 ± 0.01 Å), which have the largest peptide number asymmetry δ and largest area mismatch. This small difference in thickness is in good agreement with an earlier study by Park et al. (2), which examined the effects of area mismatch in lipid bilayers and found only modest thinning (<1 Å) of pure POPC bilayers, even up to a 25% area mismatch between leaflets.

The effects of area stress show themselves more clearly when examining the minimum thickness and deviation within the leaflet position (Table S2), which are indicative of lateral thickness fluctuations and membrane undulations. For all three pairs of matched systems, the AS piscidin distribution leads to a smaller minimum thickness and larger magnitude of leaflet deviations than the comparable AR piscidin distribution. The effect is most pronounced in AS 10/0 and AR 5/5, which have a peptide asymmetry of $\delta = 10$ and the largest area mismatch (33.2 %); the minimum bilayer thickness for AS 10/0 (16.62 ± 0.01 Å) is 1.5 Å thinner than for AR 5/5 (18.16 ± 0.01 Å), and leaflet deviations are ~2 Å larger (-9.06 ± 0.02 Å compared to -7.01 ± 0.01 Å).

Earlier studies of piscidin demonstrated that it is primarily surface bound, forming transient funnel-like defects rather than stable transmembrane pores (1, 3, 4). For all six systems simulated, defects were identified as a location within the bilayer where there is both significant thinning and significant invagination of one leaflet (see Methods); many defects were detected for only a single saved frame of the trajectory, though some defects persisted over many frames and could therefore be assigned a length based on the simulation time between the first and final detection of that defect. Table S7 lists the number of defects, defect rates (defects/µs), average defect lengths, percentage of the simulation for which a defect was present, and leaflet distribution of defects.

In all three pairs of matched systems, the defect rate is significantly higher with the AS distribution than with the corresponding AR distribution (Figure S2). As with the bilayer properties discussed above, the differences are most distinct when comparing AS 10/0 and AR 5/5, as these systems represent the δ and largest area mismatch. Defects in AS 10/0 are substantially more frequent, with a defect rate of 17.6 µs^{-1} compared to 0.7 µs^{-1} in AR 5/5. Furthermore, while all of the defects in AR 5/5 were detected for only a single frame, many of the defects in AS 10/0 persisted over multiple frames, giving an average defect lifetime of 1.87 ± 0.36 ns.

For the AR systems, there is no statistical difference in defect rate or likelihood when comparing the 3/3 and 5/5 peptide distributions, while the defect rate is slightly higher in the 7/7 distribution (2.1 µs^{-1}). In these three systems, which are symmetric, the defects are observed to form in either leaflet with roughly equal likelihood (Table S7). In the AS systems, however, defects almost exclusively originate in the leaflet with excess area (i.e. the top leaflet). This is not surprising, given that most or all of the peptides are located in the top leaflet for the AS systems. Furthermore, leaflet invagination, which is a prerequisite for defects as defined in this work, represents one way of increasing the leaflet's surface area without changing the cross-sectional area of the simulation box, meaning it should be more favorable in the leaflet with excess area.

The free energy profiles calculated in Figure 3 can be used to estimate the energy of defect formation. There is no a priori correspondence between defects and values of ξ since the two were calculated using different definitions. However, when ξ values were calculated for all defect frames, the average ξ was 0.47 ± 0.02 for all six systems (Table S6). Using this value to define a defect, the approximate energies of defect formation are 3.7 kcal/mol for AR 5/5, 3.0 kcal/mol for AR 7/7, 2.3 kcal/mol for AS 10/5, and 1.6 kcal/mol for AS 10/0, correlating with the defect rates. An alternate way to estimate the energy of defect formation is with the relative defect likelihoods from the unbiased simulations, listed in Table S7. The energies estimated by these two methods do not give identical values, likely due to the somewhat arbitrary nature of both definitions; however, the calculated energies demonstrate good correspondence between the two definitions (Figure S5), with a proportionality constant of 1.39. The excellent linear fit of the data indicate that it might be possible to use defect rates calculated from unbiased simulations to predict information about the poration energy landscape.

S1.2 Consequences of lipid translocation and area stress on free energy estimation. Due to the rapid lipid translocation that occurs in the AS simulations, both during the pore opening simulations and the umbrella sampling windows, free energy profiles calculated for these systems do not capture the full effects of area stress and likely overestimate the poration energy. In particular, the larger the value of ξ (or the pore radius), the more quickly and easily lipid translocation occurs. This means the overestimation of the free energy profile is greatest for large values of ξ, and that free energy profiles are most reliable for values of $\xi \lessapprox 1$ where no lipid translocation occurs.

The effects of this partial asymmetry loss can be estimated by comparing AS 10/0 to a similar system, with 10 P1 in one leaflet but where the lipids have been redistributed between leaflets to relax the area stress (referred to as AR 10/0 in Paper 1). In this system, poration is significantly less favorable than in AS 10/0, by about 5 kcal/mol over the entire ξ range (Figure S3). The only difference between these two systems is in the lipid distribution between leaflets – 133/167 for AR 10/0 and 144/156 for AS 10/0 (average distribution at the end of the pore opening simulations). If the free energy is assumed to scale linearly with the lipid distribution, then the poration energy of the fully area-stressed system (with a 150/150 lipid distribution), would be about 2.5 kcal/mol lower than the AS 10/0 free energy reported in Figure 3. Notably, the AR 10/0 free energy landscape is

remarkably similar to that of AR 5/5 for pore initiation (ξ <1.0), and only starts to deviate at larger ξ values. This direct comparison of AS 10/0, AR 10/0, and AR 5/5 free energy profiles in Figure S3 demonstrates that area stress, not peptide asymmetry per se, accounts for most of the differences between the different systems studied here.

S1.3 Correlation of defect energy when calculated from unbiased and biased simulations. The free energy profiles can be used to estimate the energy of defect formation. There is no a priori correspondence between defects and values of ξ since the two use different definitions. However, when ξ values were calculated for all defect frames, the average ξ was 0.47 ± 0.02 for all six systems (Table S6). Using this value to define a defect, the approximate energies of defect formation are 3.7 kcal/mol for AR 5/5, 3.0 kcal/mol for AR 7/7, 2.3 kcal/mol for AS 10/5, and 1.6 kcal/mol for AS 10/0, correlating with the defect rates. An alternate way to estimate the energy of defect formation is with the relative defect likelihoods from the unbiased simulations, listed in Table S7. The energies estimated by these two methods do not give identical values, likely due to the somewhat arbitrary nature of both definitions; however, the calculated energies demonstrate good correspondence between the two definitions (Figure S5), with a proportionality constant of 1.39. The excellent linear fit of the data indicate that it might be possible to use defect rates calculated from unbiased simulations to predict information about the poration energy landscape.

S2 Supplementary Materials and Methods

S2.1 Peptides. Purification was performed using reverse phase High Performance Liquid Chromatography using a water/acetonitrile gradient supplemented with 0.1% trifluoroacetic acid (TFA) (5). Following lyophilization, the peptide was dissolved in dilute HCl and lyophilized extensively to remove the residual TFA (1, 4, 6, 7). Any excess salt was then removed by dialysis with 1.0 kD molecular weight cut-off bags (Repligen, Waltham, MA). After reconstituting the peptides in water and adjusting the pH to 7.4, amino acid analysis was performed at the Protein Chemistry Lab at Texas A&M University (College Station, TX) to verify the amino acid content of each peptide and characterize the concentration of each solution.

S2.2 Preparation of large unilamellar vesicles. For the requenching assay, the procedure published by Wimley et al. (8–10) was followed, with some minor modifications. LUVs were prepared using 4 µmol of lipids at the indicated composition. Aggregation of anionic lipids by cationic peptides has been found to be inhibited by presence of PEG-2k lipid (11–13). Thus, PEG-2k was added in a quantity equal to 2% of the 4 µmol. After co-dissolving the lipids, the chloroform was evaporated with nitrogen gas and each film lyophilized overnight. Next, the film was hydrated with 0.5 mL of 12 mM ANTS dye and 48 mM DPX quencher dissolved in degassed 4-(2-hydroxyethyl)-1-piperazineethanesulfonic acid (HEPES) buffer (50 mM, 100 mM NaCl, 3 mM EDTA, 0.01% w/v NaN3, pH 7.4). 5 freeze-thaw cycles were then performed before extruding the suspension 23 times through a 0.1 µm polycarbonate membrane filter (Whatman, Florham Park, NJ). Next, the vesicles were run through a Sephadex G-50 column (GE Healthcare, Pittsburgh, PA) to remove the excess ANTS and DPX. Since the vesicles were not visible to the naked eye as they proceeded through the column, fractions of equal volumes were collected. Two aliquots from each fraction were taken and diluted 25-fold in a well-plate. Triton X-100 was added to reach a concentration of 0.48% in the wells. A buffer-only treatment was used as the negative control. Unless otherwise indicated, the fluorescence of the ANTS dye was measured using a SpectraMax iD5 plate reader (Molecular Devices, San Jose, CA), with excitation at 380 nm and emission at 520 nm. The most concentrated vesicles were identified as the fractions having the highest fluorescence. Their concentration was determined using a total phosphorus quantification assay (14). Dilution to 56 µM was performed before completing the assays described below.

S2.3 Start-stop dye leakage assay. For each lipid system, calcein-loaded LUVs of each lipid system were plated in triplicate at a concentration of 56 µM in HEPES buffer and monitored for 10 minutes at 20 °C on a BioTek H4 Synergy Hybrid Microplate Reader (Agilent, Santa Clara, CA) to verify their stability. They were then treated with peptide at four concentrations bracketing the half maximal effective concentration (EC_{50}) of P1. L/P = 478, 239, 119, and 60 were used for all lipid systems to enable direct comparison. Each L/P sample was plated in triplicate. The positive and negative controls were vesicles treated with Triton-X 100 and buffer, respectively, with at least two wells used for normalization.

For each time point that a measurement was made, the fractional leakage was calculated:

$$\text{Fractional leakage} = \frac{I_x - I_{\text{buffer}}}{I_{\text{triton}} - I_{\text{buffer}}} \qquad [1]$$

where I_{buffer} and I_{triton} are the fluorescence of the negative and positive controls, respectively, and I_x is the fluorescence of the measured sample. Since each sample was plated in triplicate, the averages were calculated and plotted with their standard deviations as error bars. The final graphs were made in GraphPad Prism (GraphPad, San Diego, CA).

To estimate the rates of the fluorescence bursts at each L/P, a single well measurement was performed and repeated to obtain duplicates. An analysis script was written in MATLAB (Mathworks Inc, Natick, MA). The time course data from the first few minutes post-peptide addition was imported from MS Excel, and first derivatives of the fluorescence were calculated from the first 10 sec of readings. Standard errors of the mean were calculated from the duplicate measurements.

S2.4 High throughput fluorescence requenching assay. The protocol for the leakage assay developed by White and colleagues (9, 10) was adapted to a 96-well plate setup to provide a high throughput setup. The peptide was diluted from a common stock to generate a range of L/P ratios: L/P ratios = 239, 119, 60, 30, 15, 13, 11.2, 9.3, 7, and 5.6 in 3:1 POPC:POPG, and 956, 478, 239, 119, 60, 30, 15, and 7 in 2:1:1 POPC:POPG:lysoPC. In all wells, 180 µL of LUVs were applied. Samples A corresponded to lipids treated with 20 µL of 4.8% Triton X-100 (to yield 0.48% total concentration in the wells); Samples B featured lipids

treated with 20 µL of peptide (to yield the indicated L/P); and Samples C corresponded to LUVs treated with 20 µL of buffer. Samples A were used to report on the state of the lipids when the maximum possible leakage has occurred. From Samples C, the amount of unencapsulated dye in the wells could be deduced. All 96-well plates were incubated for 1 hr at 27 °C in a shaking incubator. To quantify the amount of internal quenching, the external dye was titrated using four additions of 90 mM DPX, 5 µL each. A final addition of 4.5 µL HEPES buffer was made to Sample A while Samples B and C received 4.5 µL of 24% Triton X-100 (to yield a final well concentration of 0.48%). Notably, at the end point of the titrations done to Samples A, B, and C, the wells are expected to produce the same fluorescence since they contain the same amounts of positive control and quencher.

The experimental data were analyzed to plot Q_{in} vs f_{out} and determine the mode of leakage across the vesicular membrane, as previously described (9, 10). The measurable quantity is the total fluorescence F, which is the sum of F_o and F_i, the fluorescence outside and inside the LUVs, respectively. F_{max} is the maximum possible amount of fluorescence obtained if there were no quenching. In Sample A, where the LUVs were treated with Triton X-100, the DPX that was trapped in the vesicles becomes infinitely diluted in the buffer, enabling ANTS to fluoresce fully and yield F_{max}. By defining the quenching outside of the LUVs as Q_{out}, inside as Q_{in}, and the total quenching as Q_{total}, the following relationships can be obtained:

$$Q_{\text{out}} = \frac{F_o}{F_o^{\text{max}}} \quad [2a]$$

$$Q_{\text{in}} = \frac{F_i}{F_i^{\text{max}}} \quad [2b]$$

$$Q_{\text{total}} = \frac{F}{F^{\text{max}}} \quad [2c]$$

Based on these definitions, Q values of 1 correspond to the absence of quenching (maximal fluorescence). Equations 2a-2c can be used to solve for the total fluorescence F:

$$F = Q_{\text{total}} F_{\text{max}} = Q_{\text{out}} F_o^{\text{max}} + Q_{\text{in}} F_i^{\text{max}} \quad [3]$$

To describe the fraction of ANTS outside and inside the LUVs as a function of the fraction of dye outside (f_{out}) and inside (f_{in}) the vesicles, the following equations were implemented:

$$f_{\text{out}} = \frac{F_o}{F^{\text{max}}} \quad [4a]$$

$$f_{\text{in}} = \frac{F_i}{F^{\text{max}}} \quad [4b]$$

$$f_{\text{out}} + f_{\text{in}} = 1 \quad [4c]$$

The total quenching from eq 3 can then be expressed as:

$$Q_{\text{total}} = Q_{\text{out}} f_{\text{out}} + Q_{\text{in}}(1 - f_{\text{out}}) \quad [5]$$

From this equation, Q_{in} and f_{out} are needed to generate the Q_{in} versus f_{out} plots. The analysis was done in MS Excel (Microsoft, Redmond, WA). All fluorescence values were corrected for background and dilution by subtracting the signal from wells containing only buffer and multiplying by the total volume of solution in the wells. Q_{out} values were obtained from Sample A by calculating $F_{\text{DPX}}/F^{\text{max}}$, where F_{DPX} is the fluorescence of each well that received DPX and F^{max} is the fluorescence of Sample A before adding any DPX. Q_{total} values were obtained from Samples B and C by dividing the measured fluorescence by F^{max} from Sample A. From the linear fits obtained by plotting Q_{out} versus Q_{total}, a line following eq 5 was obtained, with f_{out} as the slope and $Q_{\text{in}}(1 - f_{\text{out}})$ as the y-intercept. The coefficient of determination R^2 from these plots was used to evaluate the goodness of the fits. The values from Sample B were corrected for incomplete entrapment using the data collected for Sample C and the following equation:

$$f_{\text{out}}^{\text{corrected}}[\text{Sample B}] = \frac{f_{\text{out}}[\text{Sample B}] - f_{\text{out}}[\text{Sample C}]}{1 - f_{\text{out}}[\text{Sample C}]} \quad [6]$$

Each Q_{in} versus f_{out} plot was built using the final values of Q_{in} versus f_{out} from the series of Sample B, collected at varying L/P ratios.

S2.5 Conventional Molecular Dynamics simulations. Conventional MD simulations were run with OpenMM version *7.4.1* (15) and Anton 2 software version *1.57.1c7* (16). Systems were first equilibrated for 250 ns in OpenMM before being moved to Anton 2 for production MD. Production simulations were carried out for 5 µs on Anton 2. Due to limited Anton 2 time, systems were then simulated for an additional 5 µs in OpenMM, for a total of 10 µs of production MD per system.

OpenMM simulations utilized the Nosé-Hoover chain (17–19) velocity Verlet integrator implemented in *openmmtools* (20) and the Monte-Carlo membrane barostat (21). Bonds with hydrogen were constrained using the SETTLE and CCMA algorithms (22, 23). A 12.0 Å cutoff was used, with a force-switching function from 8-12 Å. Long-range electrostatics were treated using the particle mesh Ewald method (24). Simulations on Anton 2 utilized the Multigrator framework (25), the Nose-Hoover thermostat (310 K), and the semi-isotropic MTK barostat. An 8 Å cutoff distance was used, and long-range electrostatics were evaluated with the u-series method (16).

S2.6 Pore opening and Umbrella Sampling simulations. All GROMACS simulations utilized a 1.2 nm cutoff distance with force switching from 0.8–1.2 nm. Bonds with hydrogen were constrained using the LINCS algorithm (26). The temperature was controlled at 310 K using velocity rescaling ($\tau = 0.5$ ps), with the water, lipids, and peptides coupled to separate baths (27). In most simulations, the pressure was controlled at 1 bar using Parrinello-Rahman barostat (28, 29) with $\tau = 5$ ps; the only exceptions were the pore opening simulations, which utilized the semi-isotropic Berendsen barostat (30) for its greater numerical stability. Long-range electrostatics were treated using the particle mesh Ewald method (24).

For simulations utilizing the extended pore reaction coordinate ξ the following parameters were used: 0.8 nm cylinder radius, 29 slices taken along the z-axis, and a slice thickness of 0.1 nm; these yielded a reference radius $R_0 = 0.422$ nm. A pore height of 1.25 nm was used for calculating the pore radius, and a switching value of $\xi = 0.925$ was used for smoothly switching between the chain and radius definitions of ξ. The polar atom definition included the oxygen atoms of water molecules and lipid phosphate groups; consistent with previous work by Verbeek et al. (31), peptide atoms were not included in the pore definition so that the definition of ξ would be identical between systems and peptides would not be unduly biased to the pore. The lateral position of the transmembrane cylinder was fixed when $\xi < 0.7$ to avoid integration problems arising from rapid lateral movements of the cylinder due to water fluctuations within the bilayer headgroup region (32). A weak half harmonic restraint was placed on the bilayer C3 atoms, with a force constant of 100 kJ/mol/nm^2 and active only when >2.1 nm from the bilayer midplane; this allowed for natural fluctuations of lipid headgroups but prevented very large membrane undulations from forming, which is necessary when $\xi > 1$.

In the pore opening simulations, ξ was slowly increased from 0.2 to 3.25 over 125 ns, with the initial nucleation from 0.2 to 1.0 taking place over 50 ns and expansion from 1.0 to 3.25 taking place over 75 ns, with a force constant of 3000 kJ/mol. Five replicates were generated for each system, with defect frames from the unbiased simulations used as starting coordinates. To ensure the pore formed at the defect locus, the lateral position of the transmembrane cylinder was fixed to the position of the defect when $\xi \leq 1.0$. Frames from each of these five replicates were then used as initial coordinates for the umbrella sampling windows.

For each PMF, 36 windows were simulated ranging from $\xi = 0.065$ to 2.93: 0.065 to 0.625 in increments of 0.08; 0.68 to 1.10 in increments of 0.03; and 1.13 to 2.93 in increments of 0.15. The force constants used were 3000 kJ/mol for $\xi < 0.65$; 5000 kJ/mol for $0.65 < \xi < 1.12$; and 500 kJ/mol for $\xi > 1.12$. Each window was simulated five times, with each replicate starting from one of the five pore opening simulations and a simulation length of 225 ns per replicate; the first 25 ns were removed as equilibration. This yielded a total of 1 µs of sampling per window, and 36 µs in aggregate per PMF. Because lipids and peptides readily translocate through the pore, especially in AS systems, five shorter replicates were performed rather than one long simulation in an attempt to avoid time-varying changes in the PMF as asymmetry was lost.

S2.7 Molecular Dynamics simulation analysis. To calculate the extent of area mismatch between leaflets in the AS systems, the cross-sectional area of a single P1 was estimated to be ∼466 Å^2 and the equilibrium dimensions for a peptide-free 2:1:1 POPC:POPG:lysoPC bilayer with 150 lipids per leaflet was calculated to be 96.8×96.8 Å^2 (or 9364 Å^2). Percent area mismatch ϕ was defined as in Park et al. (2) to be the difference in equilibrium area between the top and bottom leaflets, relative to the top (larger) leaflet:

$$\phi = 1 - \frac{A_\mathrm{T}}{A_\mathrm{B}} = 1 - \frac{N_{\mathrm{l_t}} A_{\mathrm{l_t}} + N_{\mathrm{p_t}} A_{\mathrm{p_t}}}{N_{\mathrm{l_b}} A_{\mathrm{l_b}} + N_{\mathrm{p_b}} A_{\mathrm{p_b}}} \qquad [7]$$

where A_T and A_B are the equilibrium areas of the top and bottom leaflets, calculated from N_l and N_p, the number of lipids and peptides in the leaflet, multiplied by the equilibrium cross-sectional area of a single lipid or peptide, A_l and A_p. Using $A_\mathrm{l} = 62.4$ Å^2 and $A_\mathrm{p} = 466$ Å^2 yields area mismatches of 33.2% for AS 10/0, 19.9% for AS 5/0, and 16.6% for AS 10/5. AR systems are symmetric and have no area mismatch.

In pore simulations, lipid and peptide translocations were assessed by tagging peptides and lipid headgroups based on which leaflet they were originally placed in when the systems were constructed, and monitoring their positions over the course of the simulations; the center of mass position of the lipid headgroups or peptides relative to the bilayer midplane were used to assign them to a given leaflet. Peptide insertion depth was determined by calculating the center of mass position of the backbone of the N- and C-terminal residues with respect to the bilayer midplane. The bilayer midplane was estimated using the terminal methyl groups of the lipids.

S2.8 Requenching Assay Modeling. The basic equation utilized in fluorescence requenching experiments relates the degree of internal quenching, Q_in, to the fraction of dye released from the vesicles, f_out (9):

$$Q_\mathrm{in} = [(1 + K_d \cdot [\mathrm{DPX}]_0 \cdot (1 - f_\mathrm{out})^\alpha) \cdot (1 + K_a \cdot [\mathrm{DPX}]_0 \cdot (1 - f_\mathrm{out})^\alpha)]^{-1} \qquad [8]$$

where Q_in and f_out are defined as in eqs 2b and 4a, $[\mathrm{DPX}]_0$ is the initial DPX concentration prior to any leakage, and K_d and K_a are constants that describe the dependence of quenching on the DPX concentration with $K_d = 50$ M^{-1} and $K_a = 490$ M^{-1} for ANTS/DPX (9). The parameter α accounts for the possibility of preferential DPX or ANTS release, and is the ratio of their respective release rates: $\alpha = k_\mathrm{DPX}/k_\mathrm{ANTS}$. When $\alpha = 1$, it means ANTS and DPX are released with equal likelihood. In requenching experiments, the above quantities are averaged over all vesicles in the sample and the variables in eq 8 are interpreted as bulk quantities, i.e. f_out is the fractional leakage out of all vesicles and Q_in is determined from the internal fluorescence of all vesicles. However, the equation can be applied as well to a single vesicle, where Q^v_in and f^v_out are used to denote the internal quenching and fractional leakage of a single vesicle.

The response of a single vesicle at a given c_p is simulated in two steps. First, a random number r_N between 0 and 1 is sampled; if $r_\mathrm{N} > p_\mathrm{leak}(c_\mathrm{p})$, the vesicle does not leak and $f^v_\mathrm{out} = 0$ by definition, and if $r_N \leq p_\mathrm{leak}(c_\mathrm{p})$, the vesicle is said to have leaked. For a vesicle that leaks, the extent of leakage f^v_out is determined stochastically by sampling from a Gaussian distribution with a mean of $\langle f_\mathrm{out}(c_\mathrm{p})\rangle$and standard deviation σ. The rationale for sampling from a distribution is that experimental statistics of dye leakage into individual GUVs reveal broad distributions in leakage degree across the GUV population, regardless of whether the leakage is graded or all-or-none (33–35). Q^v_in is then calculated from f^v_out using eq 8. This process is repeated N times to simulate N vesicles at a given c_p, with $\langle Q_\mathrm{in}\rangle_{c_\mathrm{p}}$ and $\langle f_\mathrm{out}\rangle_{c_\mathrm{p}}$ determined by averaging over all N of the Q^v_in and f^v_out. This simulates a single "experiment" and represents one point along the requenching response curve; the above process is repeated over a broad range of c_p to generate the full requenching response curve.

Equation 8 accounts only for the initial concentration of DPX, and has a maximal value when $f_\mathrm{out} = 1$. In practice, however, $f^v_\mathrm{out} = 1$ indicates that all of the ANTS has leaked out of the vesicle, rendering it invisible to the experiment. Furthermore, when *most* of the ANTS has leaked out of a given vesicle, the signal will be negligibly small when compared to the contribution to F^{max}_{in} from vesicles that are still mostly or completely full. For this reason, for any vesicle with $f^v_\mathrm{out} > 0.9$, Q^v_in was set to NaN (Not A Number). The $f_\mathrm{out} < 0.9$ cutoff is consistent with the range considered to be measurable experimentally (9, 10) as well as a similar cutoff used in a related leakage model (35).

$p_\mathrm{leak}(c_p)$ and $\langle f_\mathrm{out}(c_\mathrm{p})\rangle$ were assumed to be sigmoid functions of c_p, of the form:

$$f(c_\mathrm{p}) = \frac{1}{2} \cdot \tanh[C_1 \cdot (c_\mathrm{p} - C_2)] + \frac{1}{2} \quad [9]$$

C_1 controls how steeply the curve rises, and C_2 shifts the midpoint along c_p. Sigmoid functions were selected because experimental measures of dye leakage from vesicle populations as a function of peptide concentration are often sigmoidal (1, 36–38). Additionally, both $p_\mathrm{leak}(c_\mathrm{p})$ and $\langle f_\mathrm{out}(c_\mathrm{p})\rangle$were allowed to take constant values in the range [0,1]. All modeled data presented here utilized $\sigma = 0.025$ for the f^v_out sampling Gaussian distribution, $N = 50{,}000$ simulated vesicles per c_p, and 100 c_p values ranging from 0.01 to 1.00 in increments of 0.01. In general, $\alpha = 1$ was assumed, unless specifically noted otherwise.

S3 Abbreviations and Symbols

AMP – antimicrobial peptide
ANTS – fluorescent dye, 8-aminonaphthalene-1,3,6-trisulfonic acid
AR – area-relaxed
AS – area-stressed
CPP – cell-penetrating peptide
DPX – quencher, p-xyleen-bis-pyridinium bromide
EC_{50} – half maximal effective concentration
lysoPC – lysophosphatidylcholine
LUV – large unilamellar vesicle
L/P – lipid-to-peptide ratio
MAP – membrane-active peptide
MD – molecular dynamics
P1 – piscidin 1
PMF – potential of mean force
POPC – 1-palmitoyl-2-oleoyl-glycero-3-phosphocholine
POPG – 1-palmitoyl-2-oleoyl-sn-glycero-3-phosphoglycerol

C_1, C_2, C_3, C_4 – fit parameters for unified model
c_p – peptide concentration, arbitrary units with range [0,1]
$[DPX]_0$ – in requenching assay, initial DPX concentration prior to any leakage
f_{out} – in requenching assay, fraction of released ANTS
$\langle f_\mathrm{out}\rangle$ – fractional leakage of a vesicle
F(ξ) – poration free energy as a function of ξ
p_leak – probability of a vesicle leaking
Q_{in} – in requenching assay, normalized fluorescence of ANTS inside the vesicles
α – ratio of DPX and ANTS release rates ($\alpha = k_\mathrm{DPX}/k_\mathrm{ANTS}$)
δ – peptide number asymmetry
ξ – extended pore reaction coordinate; values $\lesssim$1 quantify pore nucleation and values >1 quantify pore expansion

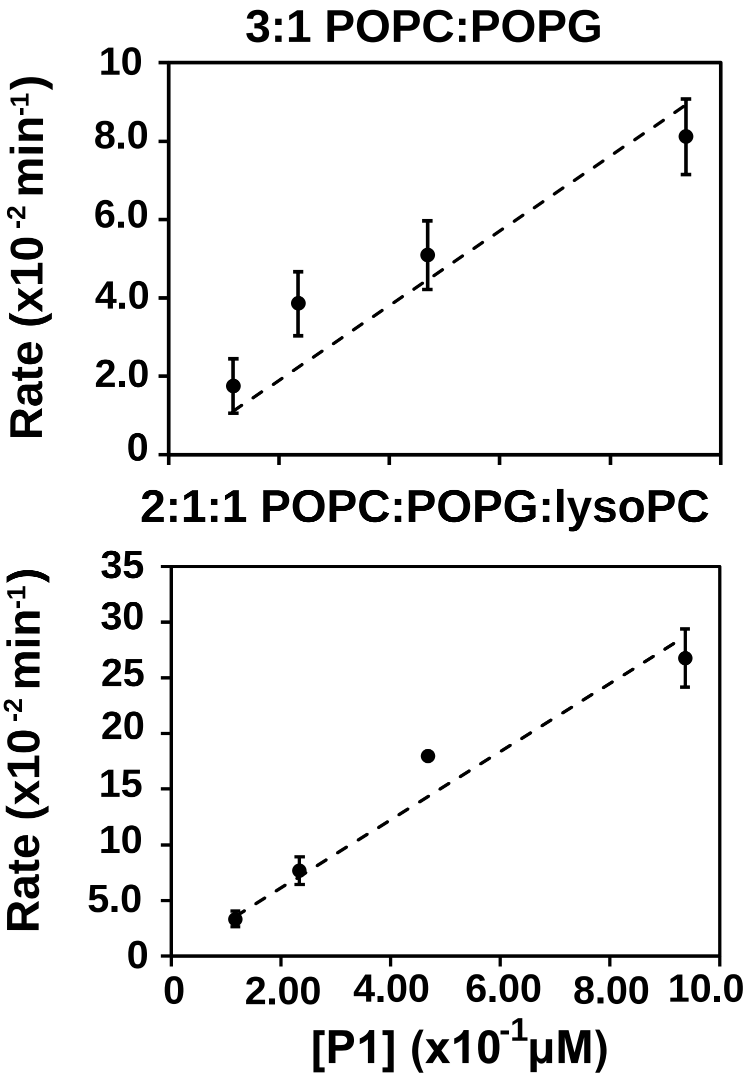

Fig. S1. Rate versus peptide concentration for the first burst of leakage following P1 addition to 3:1 POPC:POPG (top) and 2:1:1 POPC:POPG:lysoPC (bottom) LUVs. Concentration-dependent rate constants were obtained from linear fits to the data (dashed lines), and are 0.095 $\pm$ 0.014 $min^{-1} \cdot \mu M^{-1}$ for 3:1 POPC:POPG and 0.31 $\pm$ 0.02 $min^{-1} \cdot \mu M^{-1}$ for 2:1:1 POPC:POPG:lysoPC. For each lipid system, duplicates were run, with the averages and standard errors of the mean indicated on each graph. The duplicates were also fitted individually to generate individual rate constants, with the standard error of the mean representing the uncertainty on each listed rate constant.

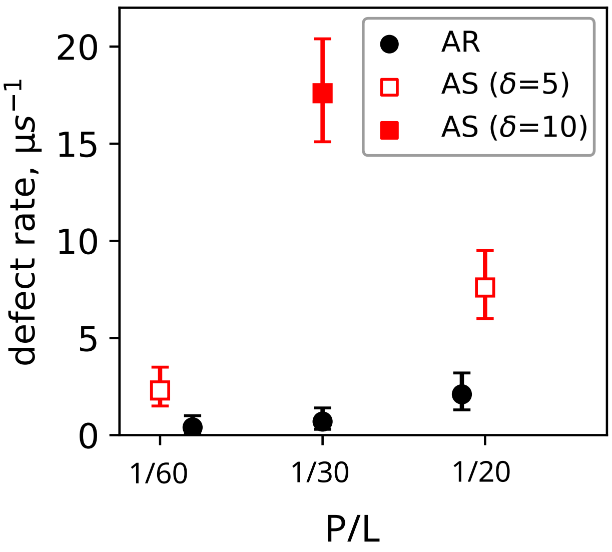

Fig. S2. Defect rates observed from all six conventional MD simulations, expressed as unique defects per μs at three different lipid-to-peptide (L/P) ratios. Black circles are area-relaxed (AR) systems with symmetric P1 distributions (3/3, 5/5, and 7/7 from left to right). Open red squares are area-stressed (AS) systems with a peptide asymmetry δ of five P1 (5/0 on the left and 10/5 on the right). The filled red square is the AS 10/0 system, where δ = 10. Error bars are 95% confidence intervals.

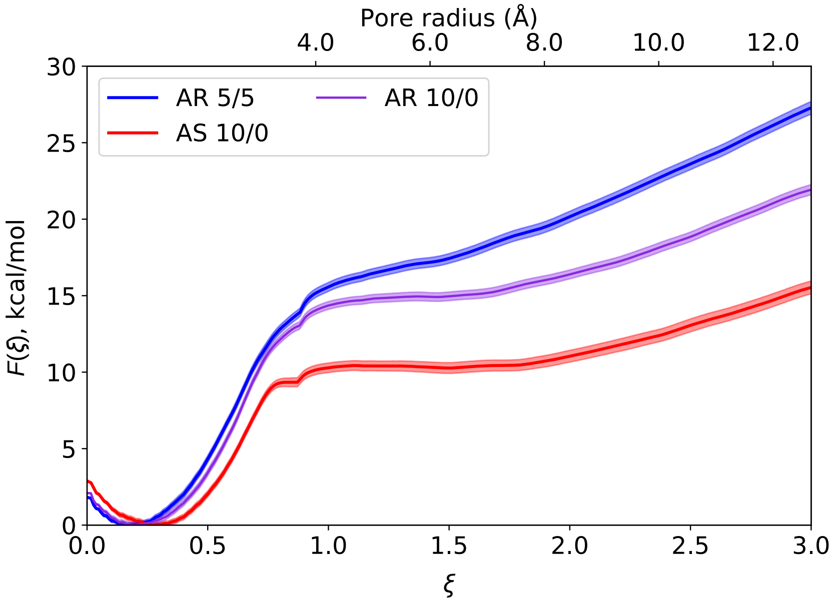

Fig. S3. Free energy $F(\xi)$ of pore formation and expansion for L/P = 30 area-stressed (AS) and area-relaxed (AR) configurations, including an area relaxed asymmetric system (AR 10/0). Values of the pore reaction coordinate ξ < 1 correspond to pore initiation, while values $\geq$ 1 correspond to pore expansion and can be converted to an approximate pore radius by multiplying ξ by 4.22 Å. Error in each free energy profile is represented by the shaded region.

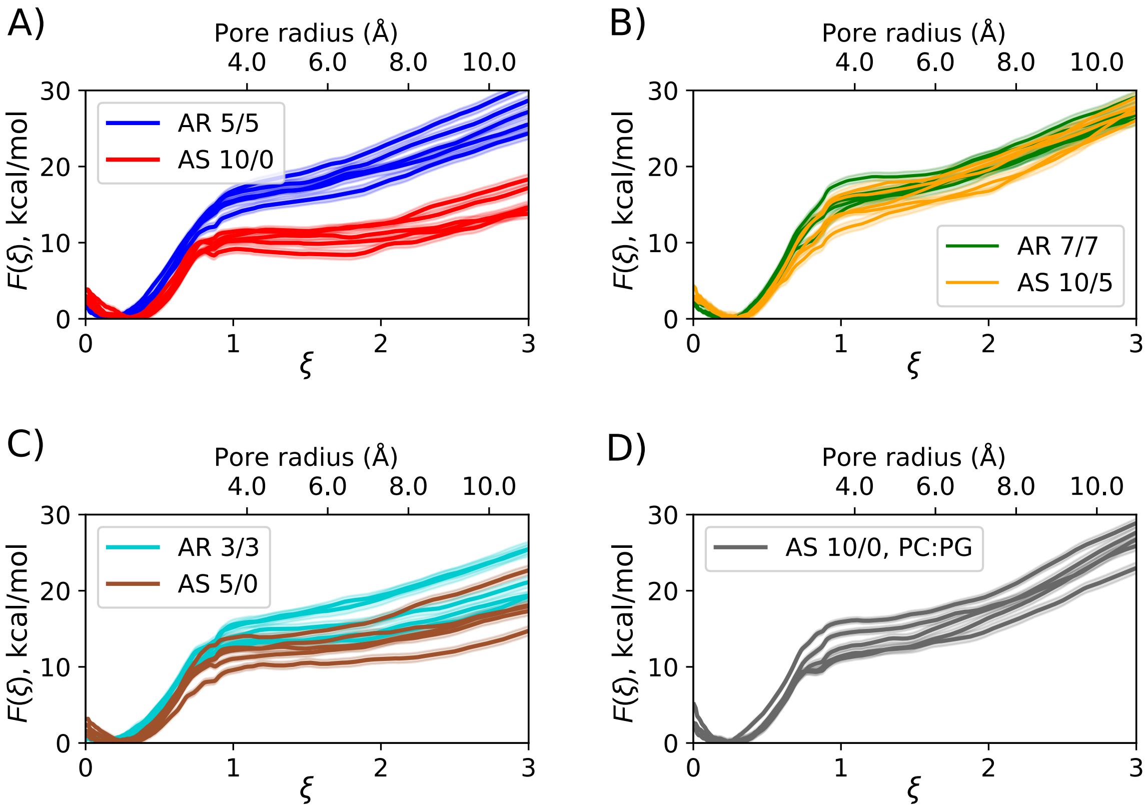

Fig. S4. Free energy $F(\xi)$ of pore formation and expansion for the five independent replicates of each area-relaxed (AR) or area-stressed (AS) system. Values of the pore reaction coordinate $\xi < 1$ correspond to pore initiation, while values ≥ 1 correspond to pore expansion and can be converted to an approximate pore radius by multiplying ξ by 4.22 Å. Error in each free energy profile is represented by the shaded region. Each curve represents an independent run for each system. (A) AR 5/5 and AS 10/0 systems. (B) AR 7/7 and AS 10/5 systems. (C) AR 3/3 and AS 5/0 systems. (D) AR 10/0 distribution in 3:1 POPC:POPG.

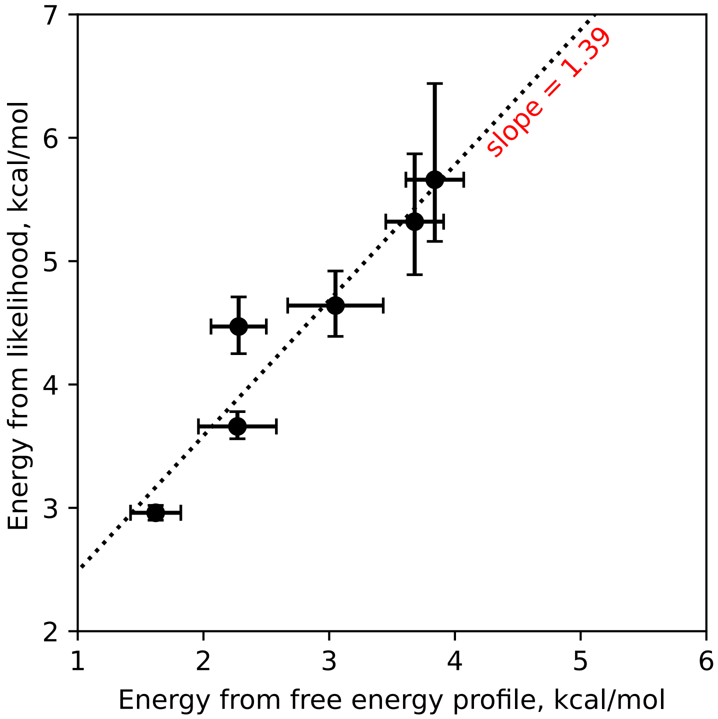

Fig. S5. Correspondence between defect energies as determined from poration free energy profiles (Table S6) and estimated from relative likelihood in the unbiased simulations (Table S7). Slope from linear fit is 1.39.

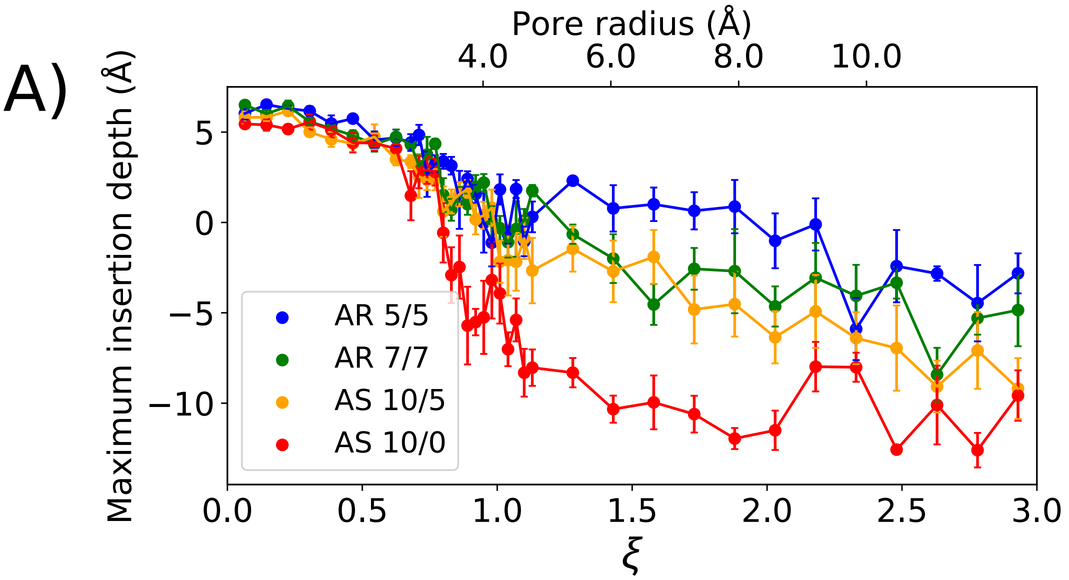

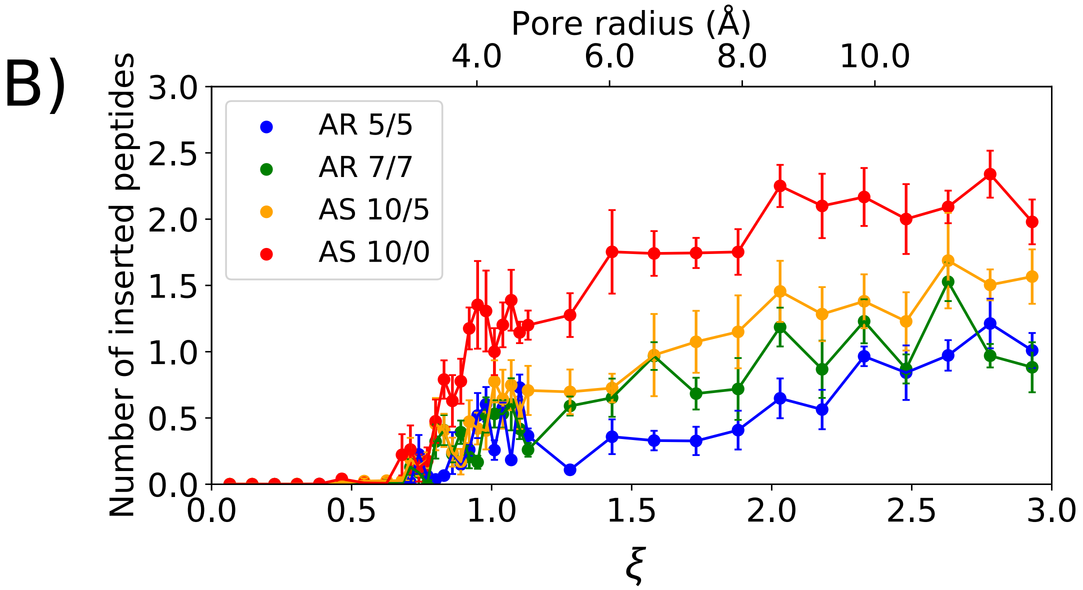

Fig. S6. A) Maximum peptide insertion as a function of the pore reaction coordinate ξ, calculated over the last 100 ns of each umbrella sampling window. A depth of 0 Å corresponds to the bilayer midplane position. B) Average number of inserted peptides as a function of ξ, calculated over the last 100 ns of each umbrella sampling window. Error bars in both panels are the standard error of the mean for the five replicates. AR - area-relaxed systems; AS - area-stressed systems.

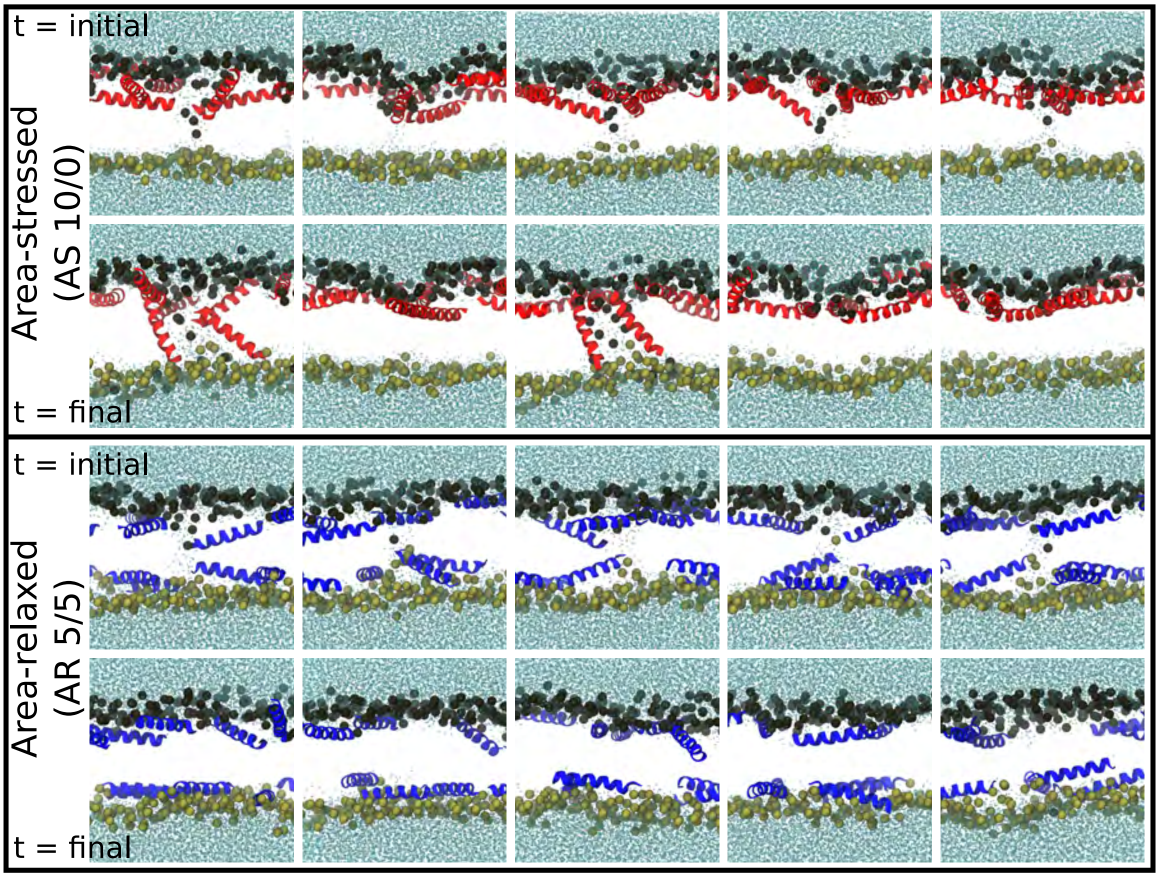

Fig. S7. Initial and final snapshots from unbiased simulations of CPP-like pores (ξ = 1.18, r = 5 Å) in area-stressed (AS) 10/0 (top panels) and area-relaxed (AR) 5/5 (bottom panels). Simulation lengths were 250 ns for AR 5/5 systems, 250 ns for AS 10/0 replicates 2, 4, and 5, and 1 µs for AS 10/0 replicates 1 and 3. Water is depicted as cyan dots, peptides are colorful helical ribbons, and lipid phosphorus atoms are spheres colored according to which leaflet they began in when the systems were constructed – black for top leaflet and gold for bottom leaflet.

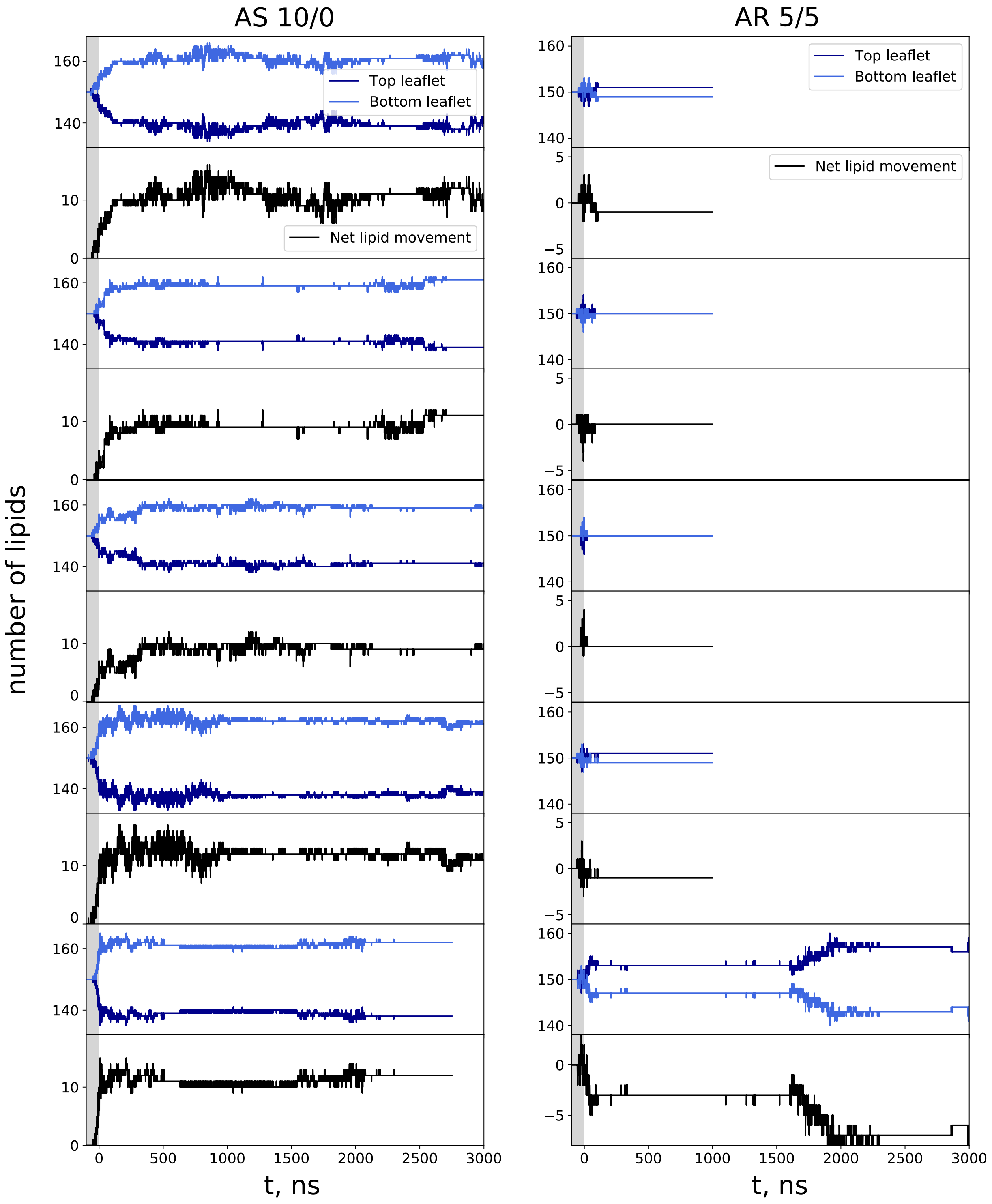

Fig. S8. Timeseries of lipids per leaflet (dark and light blue lines) and net lipid translocation (black lines) for all five replicates of the unbiased 10 Å radius pore simulations in AS (area-stressed) 10/0 (left) and AR (area-relaxed) 5/5 (right). Grey shaded regions from t = -100 to 0 ns denote the 100 ns pore opening simulations that preceded the long time scale unbiased simulations. For the four AS 5/5 replicates where pore closure occurred, the simulations were stopped after 1 µs.

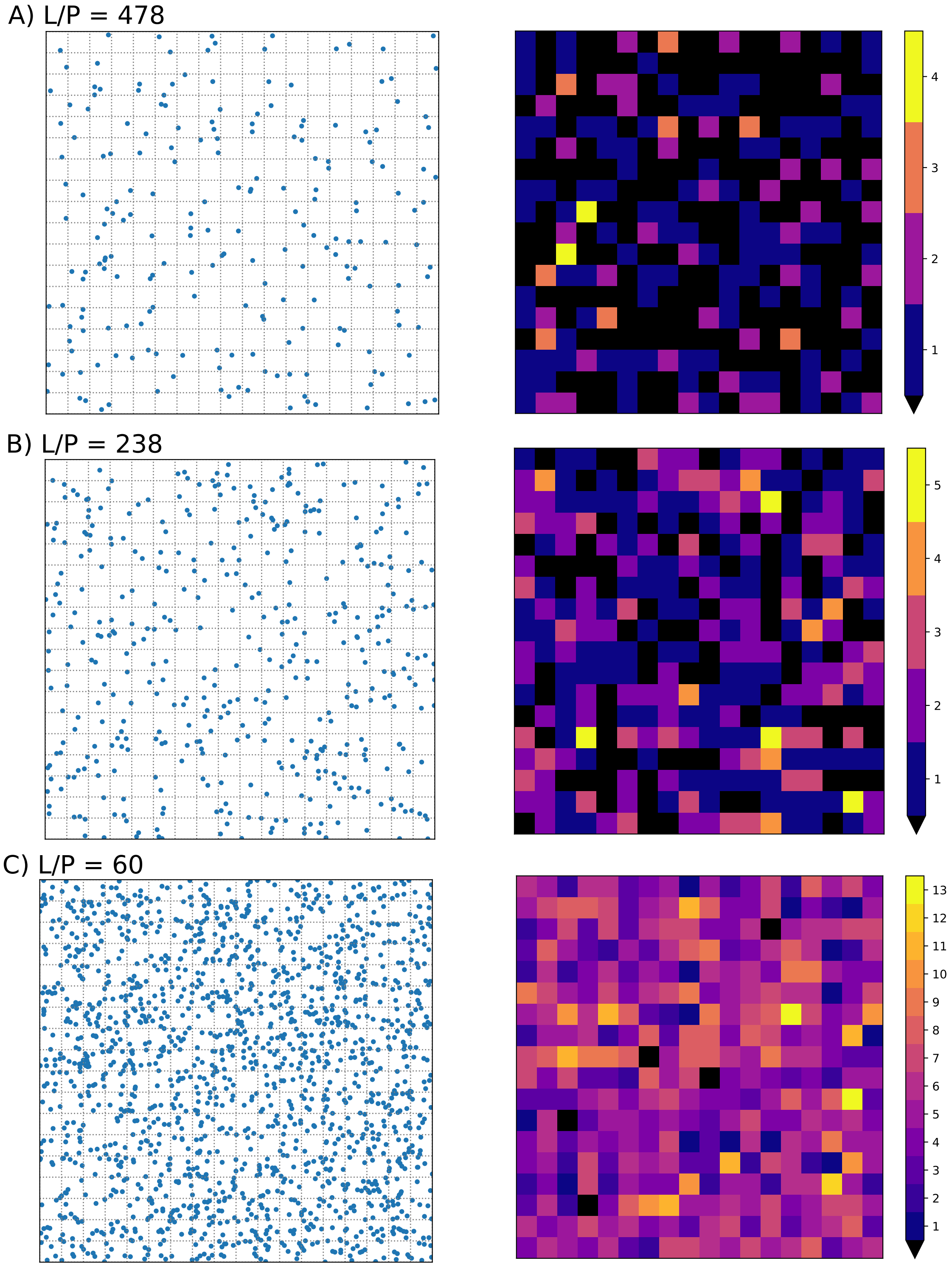

Fig. S9. Points (representing peptides) randomly distributed on an 18x18 grid modeling a 100 nm diameter vesicle subdivided into 100x100 Å grids. Simulated data for three L/P vaues: (A) 478, (B) 238, and (C) 60. Left - peptide locations indicated by dots; right - same distribution represented as a heat map of peptides per box.

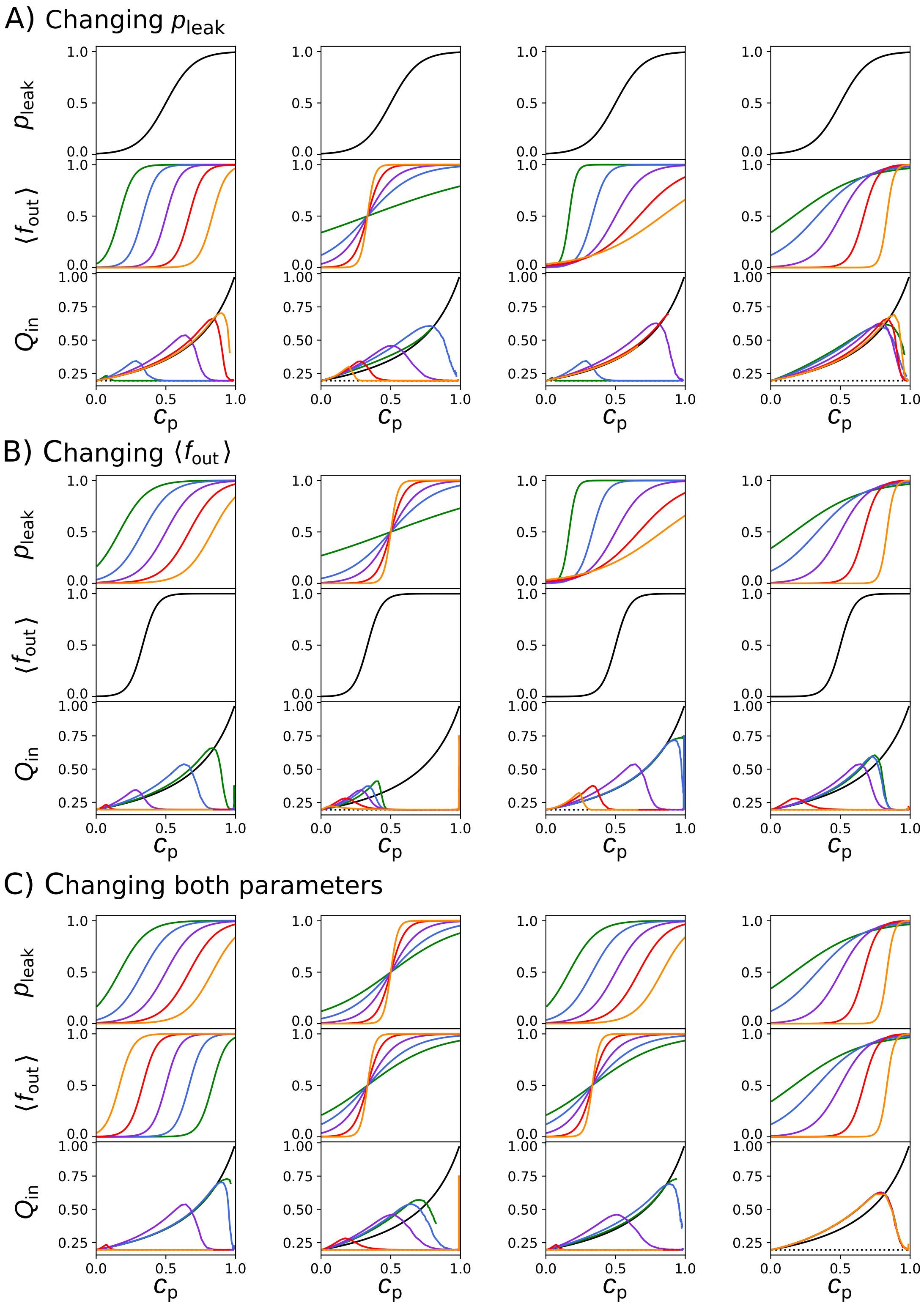

Fig. S10. Response of unified model to different choices of how the probability of leakage p_{leak} and the fractional vesicle leakage $\langle f_{\mathrm{out}} \rangle$ vary with peptide concentration c_{p}.

Table S1. EC_{50} (half maximal effective concentration) values of P1 in 3:1 POPC:POPG and 2:1:1 POPC:POPG:lysoPC LUVs. The error is reported as the standard deviation of the independent replicates of the assay.

Lipid Composition	EC_{50} (L/P)
3:1 POPC:POPG	23.6 (5.4)
2:1:1 POPC:POPG:lysoPC	119.8 (8.3)

Table S2. Average bilayer properties for all six area-stressed (AS) or area-relaxed (AR) systems simulated, along with percent area mismatch and peptide number asymmetry δ for AS systems. Control system is a peptide-free simulation, taken from Paper 1 (1). Errors reported are standard error of the mean.

Piscidin distribution	L/P ratio	Average thickness (Å)	Minimum thickness (Å)	Max leaflet deviation (Å)	Area mismatch	δ
control	—	25.57 ± 0.01	18.90± 0.02	-6.04 ± 0.02	—	—
AS 5/0	60	25.77 ± 0.01	18.23 ± 0.01	-7.61 ± 0.01	19.9%	5
AR 3/3	50	25.90 ± 0.01	18.62 ± 0.01	-6.66 ± 0.01	—	—
AS 10/0	30	24.95 ± 0.01	16.62 ± 0.01	-9.06 ± 0.02	33.2%	10
AR 5/5	30	25.60 ± 0.01	18.16 ± 0.01	-7.01 ± 0.01	—	—
AS 10/5	20	25.08 ± 0.01	17.13 ± 0.01	-8.62 ± 0.02	16.6%	5
AR 7/7	21	25.34 ± 0.01	17.74 ± 0.01	-7.48 ± 0.01	—	—

Table S3. Energies of forming a water wire, CPP-like pore, and 10 Å radius pore, as determined from the potentials of mean force. The energy differences between matched area-stressed (AS) and area-relaxed (AR) systems are given as ΔE_{ww}, ΔE_{cpp}, and ΔE_{pore}.

System	Water wire (kcal/mol)	ΔE_{ww}	CPP-like (kcal/mol)	ΔE_{cpp}	10 Å pore (kcal/mol)	ΔE_{pore}
AS 5/0	10.5 $\pm$ 0.3	–	12.0 $\pm$ 0.3	–	14.9 $\pm$ 0.4	–
AR 3/3	12.3 $\pm$ 0.3	1.8	14.7 $\pm$ 0.4	2.7	18.4 $\pm$ 0.4	3.5
AS 10/0	9.3 $\pm$ 0.3	–	10.4 $\pm$ 0.3	–	12.4 $\pm$ 0.4	–
AR 5/5	13.8 $\pm$ 0.3	4.5	16.5 $\pm$ 0.3	6.1	22.7 $\pm$ 0.4	10.3
AS 10/5	12.0 $\pm$ 0.4	–	14.9 $\pm$ 0.4	–	22.0 $\pm$ 0.5	–
AR 7/7	13.9 $\pm$ 0.4	1.9	17.3 $\pm$ 0.5	2.4	24.8 $\pm$ 0.5	2.8

Table S4. Net lipid translocation from unbiased simulations of a 10 Å radius pore initiated from an area-stressed (AS) or area-relaxed (AR) configuration, averaged over the last 250 ns of the trajectory. Positive values denote a net movement of lipids from the top leaflet to the bottom leaflet, and negative values denote movement from the bottom to the top leaflet. Error bars represent the standard error of the mean. Systems with no error given are replicates where the pore closed, fixing the final lipid translocations to their given values.

	AS 10/0	AR 5/5
Rep 1	11.0 ± 1.0	-1.0
Rep 2	11.0	0.0
Rep 3	9.1 ± 0.5	0.0
Rep 4	11.0 ± 0.3	-1.0
Rep 5	12.0	-6.5 ± 0.5
Average	10.8 ± 0.5	-1.7 ± 1.2

Table S5. Mean (N_p) and variances of peptide occupancies in 100 nm^2 patches of an LUV containing 100,000 lipids (diameter $\approx$ 100 nm).

L/P	N_p	Var (N_p)
30	10.3	10.2
60	5.1	5.1
238	1.3	1.3
478	0.6	0.6

Table S6. Average value of the pore reaction coordinate ξ for defect frames, and approximate energy of defects. Defect ξ averaged over all defect frames from each area-stressed (AS) or area-relaxed (AR) system. Defect energy is determined from the free energy profiles as the energy at ξ = 0.48.

System	Average defect ξ	Energy of defect (kcal/mol)
AS 10/0	0.51 ± 0.01	1.6 ± 0.2
AR 5/5	0.43 ± 0.03	3.7 ± 0.2
AS 10/5	0.46 ± 0.01	2.3 ± 0.3
AR 7/7	0.48 ± 0.02	3.0 ± 0.4
AS 5/0	0.44 ± 0.02	2.3 ± 0.2
AR 3/3	0.40 ± 0.08	3.8 ± 0.2

Table S7. Defect rates, average defect lifetimes, defect likelihood, defect energies, and leaflet defect ratio for all six conventional MD simulations. For defect rates, the 95% confidence interval is given in parenthesis. A defect which is present for only a single frame is assigned a length of 0. Defect energy E_{defect} calculated from defect probability. Defect likelihood is the percentage of the simulation in which defects are present. Leaflet defect ratio is the number of defects located in the top leaflet versus bottom leaflet.

Piscidin distribution	Defect rate (μs^{-1})	Average defect length (ns)	Defect likelihood	E_{defect} (kcal/mol)	Leaflet defect ratio
5/0	2.3 (1.5-3.5)	0.55 ± 0.35	0.06% (0.04-0.08%)	4.5 ± 0.3	29:0
3/3	0.4 (0.1-1.0)	0	0.01% (0.00-0.02%)	5.7 ± 0.5	1:3
10/0	17.6 (15.1-20.4)	1.87 ± 0.36	0.73% (0.65-0.80%)	3.0 ± 0.1	360:3
5/5	0.7 (0.3-1.4)	0	0.01% (0.01-0.03%)	5.3 ± 0.5	3:4
10/5	7.6 (6.0-9.5)	1.07 ± 0.30	0.23% (0.19-0.27%)	3.7 ± 0.1	109:4
7/7	2.1 (1.3-3.2)	0.02 ± 0.02	0.04% (0.03-0.07%)	4.6 ± 0.3	11:11

References

1. A Rice, et al., Investigating how lysophosphatidylcholine and lysophosphatidylethanolamine enhance the membrane permeabilization efficacy of host defense peptide piscidin 1. *The J. Phys. Chem. B* **129**, 210–227 (2025).
2. S Park, AH Beaven, JB Klauda, W Im, How tolerant are membrane simulations with mismatch in area per lipid between leaflets? *J. Chem. Theory Comput.* **11**, 3466–3477 (2015).
3. BS Perrin, R Fu, ML Cotten, RW Pastor, Simulations of Membrane-Disrupting Peptides II: AMP Piscidin 1 Favors Surface Defects over Pores. *Biophys. J.* **111**, 1258–1266 (2016).
4. M Mihailescu, et al., Structure and function in antimicrobial piscidins: Histidine position, directionality of membrane insertion, and ph-dependent permeabilization. *J. Am. Chem. Soc.* **141**, 9837–9853 (2019).
5. BS Perrin, et al., High-resolution structures and orientations of antimicrobial peptides piscidin 1 and piscidin 3 in fluid bilayers reveal tilting, kinking, and bilayer immersion. *J. Am. Chem. Soc.* **136**, 3491–3504 (2014).
6. F Comert, et al., Copper-binding anticancer peptides from the piscidin family: an expanded mechanism that encompasses physical and chemical bilayer disruption. *Sci. Reports* **11**, 12620 (2021).
7. F Liu, et al., Host defense peptide piscidin and yeast-derived glycolipid exhibit synergistic antimicrobial action through concerted interactions with membranes. *JACS Au* (2023).
8. WC Wimley, ME Selsted, SH White, Interactions between human defensins and lipid bilayers: Evidence for formation of multimeric pores. *Protein Sci.* **3**, 1362–1373 (1994).
9. A Ladokhin, W Wimley, S White, Leakage of membrane vesicle contents: Determination of mechanism using fluorescence requenching. *Biophys. J.* **69**, 1964–1971 (1995).
10. AS Ladokhin, WC Wimley, K Hristova, SH White, Mechanism of leakage of contents of membrane vesicles determined by fluorescence requenching. *Methods Enzymol.* p. 474–486 (1997).
11. X Guo, J Andrew MacKay, J Szoka, Francis C., Mechanism of ph-triggered collapse of phosphatidylethanolamine liposomes stabilized by an ortho ester polyethyleneglycol lipid. *Biophys. J.* **84**, 1784–1795 (2003).
12. WC Wimley, Determining the effects of membrane-interacting peptides on membrane integrity. *Methods Mol. Biol.* p. 89–106 (2015).
13. S Shi, H Fan, M Hoernke, Leaky membrane fusion: an ambivalent effect induced by antimicrobial polycations. *Nanoscale Adv.* **4**, 5109–5122 (2022).
14. M Pate, J Blazyk, Methods for assessing the structure and function of cationic antimicrobial peptides. *Methods Mol Med* **142**, 155–73 (2008).
15. P Eastman, et al., Openmm 7: Rapid development of high performance algorithms for molecular dynamics. *PLOS Comput. Biol.* **13** (2017).
16. DE Shaw, et al., Anton 2: Raising the bar for performance and programmability in a special-purpose molecular dynamics supercomputer in *Proceedings of the International Conference for High Performance Computing, Networking, Storage and Analysis*, SC '14. (IEEE Press, Piscataway, NJ, USA), pp. 41–53 (2014).
17. S Nosé, A molecular dynamics method for simulations in the canonical ensemble. *Mol. Phys.* **52**, 255–268 (1984).
18. WG Hoover, Canonical dynamics: Equilibrium phase-space distributions. *Phys. Rev. A* **31**, 1695–1697 (1985).
19. GJ Martyna, ML Klein, M Tuckerman, Nosé–hoover chains: The canonical ensemble via continuous dynamics. *The J. Chem. Phys.* **97**, 2635–2643 (1992).
20. J Chodera, et al., choderalab/openmmtools: 0.21.4 (2022).
21. KH Chow, DM Ferguson, Isothermal-isobaric molecular dynamics simulations with monte carlo volume sampling. *Comput. Phys. Commun.* **91**, 283–289 (1995).
22. S Miyamoto, PA Kollman, Settle: An analytical version of the shake and rattle algorithm for rigid water models. *J. Comput. Chem.* **13**, 952–962 (1992).
23. P Eastman, VS Pande, Constant constraint matrix approximation: A robust, parallelizable constraint method for molecular simulations. *J. Chem. Theory Comput.* **6**, 434–437 (2010).
24. T Darden, D York, L Pedersen, Particle mesh Ewald - an N·log(N) method for Ewald sums in large systems. *J Chem Phys* **98**, 10089–10092 (1993).
25. RA Lippert, et al., Accurate and efficient integration for molecular dynamics simulations at constant temperature and pressure. *J Chem Phys* **139**, 164106 (2013).
26. B Hess, P-lincs: a parallel linear constraint solver for molecular simulation. *J. Chem. Theory Comput.* **4**, 116–122 (2007).
27. G Bussi, D Donadio, M Parrinello, Canonical sampling through velocity rescaling. *The J. Chem. Phys.* **126** (2007).
28. M Parrinello, A Rahman, Polymorphic transitions in single crystals: A new molecular dynamics method. *J. Appl. Phys.* **52**, 7182–7190 (1981).
29. S Nose, M Klein, Constant pressure molecular dynamics for molecular systems. *Mol. Phys.* **50**, 1055–1076 (1983).
30. HJC Berendsen, JPM Postma, WF van Gunsteren, A DiNola, JR Haak, Molecular dynamics with coupling to an external bath. *The J. Chem. Phys.* **81**, 3684–3690 (1984).
31. SF Verbeek, et al., How arginine derivatives alter the stability of lipid membranes: Dissecting the roles of side chains, backbone and termini. *Eur. Biophys. J.* **50**, 127–142 (2021).
32. JS Hub, Joint reaction coordinate for computing the free-energy landscape of pore nucleation and pore expansion in lipid membranes. *J. Chem. Theory Comput.* **17**, 1229–1239 (2021).
33. B Apellániz, JL Nieva, P Schwille, AJ García-Sáez, All-or-none versus graded: Single-vesicle analysis reveals lipid

composition effects on membrane permeabilization. *Biophys. J.* **99**, 3619–3628 (2010).
34. SA Wheaten, A Lakshmanan, PF Almeida, Statistical analysis of peptide-induced graded and all-or-none fluxes in giant vesicles. *Biophys. J.* **105**, 432–443 (2013).
35. S Braun, et al., Biomembrane permeabilization: Statistics of individual leakage events harmonize the interpretation of vesicle leakage. *ACS Nano* **12**, 813–819 (2017).
36. H Heerklotz, J Seelig, Leakage and lysis of lipid membranes induced by the lipopeptide surfactin. *Eur. Biophys. J.* **36**, 305–314 (2006).
37. H Patel, Q Huynh, D Bärlehner, H Heerklotz, Additive and synergistic membrane permeabilization by antimicrobial (lipo)peptides and detergents. *Biophys. J.* **106**, 2115–2125 (2014).
38. SG Hovakeemian, R Liu, SH Gellman, H Heerklotz, Correlating antimicrobial activity and model membrane leakage induced by nylon-3 polymers and detergents. *Soft Matter* **11**, 6840–6851 (2015).
